# Spectral Clustering with Likelihood Refinement for High-Dimensional Latent Class Recovery

**DOI:** 10.1017/psy.2026.10095

**Published:** 2026-02-18

**Authors:** Zhongyuan Lyu, Yuqi Gu

**Affiliations:** 1 Columbia University, USA; 2 Department of Statistics, Columbia University, USA

**Keywords:** high-dimensional data, joint maximum likelihood estimation, latent class model, spectral clustering

## Abstract

Latent class models (LCMs) are widely used for identifying unobserved subgroups from multivariate categorical data in social sciences, with binary data as a particularly popular example. However, accurately recovering individual latent class memberships remains challenging, especially when handling high-dimensional datasets with many items. This work proposes a novel two-stage algorithm for LCMs suited for high-dimensional binary responses. Our method first initializes latent class assignments by an easy-to-implement spectral clustering algorithm, and then refines these assignments with a one-step likelihood-based update. This approach combines the computational efficiency of spectral clustering with the improved statistical accuracy of likelihood-based estimation. We establish theoretical guarantees showing that this method is minimax-optimal for latent class recovery in the statistical decision theory sense. The method also leads to exact clustering of subjects with high probability under mild conditions. As a byproduct, we propose a computationally efficient consistent estimator for the number of latent classes. Extensive experiments on both simulated data and real data validate our theoretical results and demonstrate our method’s superior performance over alternative methods.

## 1 Introduction

Latent class models (LCMs; Goodman, 1974; Lazarsfeld, 1968) are popular tools for uncovering unobserved subgroups from multivariate categorical responses, with broad applications in social and behavioral sciences (Berzofsky et al., 2014; Korpershoek et al., 2015; Wang & Hanges, 2011; Zeng et al., 2023). At the core of an LCM, we aim to identify *K* latent classes of *N* individuals based on their categorical responses to *J* items. In this article, we focus on the widely used LCMs with binary responses, which are prevailing in educational assessments (correct/wrong responses to test questions) and social science surveys (yes/no responses to survey items). Accurately and efficiently recovering latent classes in large *N* and large *J* settings remains a significant statistical and computational challenge.

Traditional approaches to LCM are typically based on maximum likelihood estimation. In the classical random-effect formulation, the latent class labels of subjects are treated as random variables, and the model is estimated by the marginal maximum likelihood (MML) approach, in which the latent labels are marginalized out. The expectation–maximization (EM) algorithm (Dempster et al., 1977) is often used for this purpose, but it becomes computationally expensive and lacks theoretical guarantees in the modern regime, where both *N* and *J* are large. In contrast, the joint maximum likelihood (JML) approach adopts a fixed-effect perspective, treating the latent class labels as unknown parameters to be estimated. However, due to the inherent non-convexity of the joint likelihood function, JML is still highly sensitive to initialization and prone to be trapped in local optima. Recently, Zeng et al. (2023) showed that JML is consistent under the large *N* and large *J* regime. Nonetheless, they do not provide any algorithmic guarantee, and the convergence rate established therein is polynomial in *J*, whose optimality remains unclear.

In this article, we consider a different approach related to spectral clustering, which leverages the intrinsic approximate low-rank structure of the data matrix and is computationally efficient. In psychometrics and related fields, the common heuristic practice for spectral clustering is to first construct a similarity matrix among individuals or items, apply eigenvalue decomposition to it, and then perform K-means clustering of the eigenspace embeddings (Chen et al., 2017; Von Luxburg, 2007). More recently, Chen and Li (2019) directly perform the singular value decomposition (SVD) on a regularized version of the data matrix itself. Similar ideas have also been developed for nonlinear item factor analysis (Zhang et al., 2020) and the grade-of-membership model (Chen & Gu, 2024).

We next explain our idea of directly adapting the SVD to perform spectral clustering for binary-response LCMs. We collect the binary responses in an N×J matrix R with binary entries, then under the typical LCM assumption, the R can be written as R=ER+E , where ER is the low-rank “signal” matrix due to the existence of *K* latent classes, and E is a mean-zero “noise” matrix. Denote by $\hat{U}\hat{\Sigma }\hat{V^{⊤}}$ the truncated rank-*K* SVD of R , then $R\approx \hat{U}\hat{\Sigma }\hat{V^{⊤}}$ approximately captures the “signal” part. In particular, we first extract $\hat{U}$ , the leading *K* left singular vectors of the data matrix to perform dimensionality reduction. Then we apply K-means clustering algorithm on the *N* rows of $\hat{U}\hat{\Sigma }$ , the left singular vectors weighted by their corresponding singular values $\hat{\Sigma }$ , to obtain the latent class label estimates. While variants of this simple yet efficient algorithm have attracted significant attention in network analysis (Zhang, 2024) and sub-Gaussian mixture models (Zhang & Zhou, 2024), it has not been widely explored or studied in the context of LCMs. The only exception is the recent work by Lyu et al. (2025), which extended spectral clustering to LCMs with individual-level degree heterogeneity and showed that spectral methods can lead to exact recovery of the latent class labels in certain regime. However, whether the spectral method gives an optimal way for estimating latent class labels in LCMs remains unknown. In this article, we resolve this question by proposing a novel two-stage method:

1. *First step:* Obtain initial latent class labels of individuals via the SVD-based spectral clustering, which leverages the approximate low-rankness of the data matrix.
2. *Second step:* Refine the latent class labels through a single step of likelihood maximization, which “sharpens” the coarse initial labels using the likelihood information.

This hybrid approach simultaneously takes advantage of the scalability of spectral methods and leverages the Bernoulli likelihood information to achieve a vanishing exponential error rate for clustering. We provide rigorous theoretical guarantees for our method. In summary, our method is both computationally fast and statistically optimal in the regime with a large number of items. Therefore, this work provides a useful tool to aid psychometric researchers and practitioners to perform latent class analysis of modern high-dimensional data. We emphasize that our method has no requirement on the magnitude of *J* compared to *N*, and even allows *J* to be much larger than *N*.

The rest of this article is organized as follows. After reviewing the setup of LCMs for binary responses in Section 2, we discuss the performance of spectral clustering and propose our main algorithm in Section 3. In Section 4, we establish theoretical guarantees and statistical optimality of our algorithm. We evaluate our method’s performance in numerical experiments in Section 5. The proofs of the theoretical results are included in the Appendix.

*Notation:* We use bold capital letters, such as A,B,⋯ to denote matrices and bold lowercase a,b,⋯ to denote vectors. For any positive integer *m*, denote m:={1,⋯,m} . For any matrix $A\in R^{N\times J}$ and any i∈N and j∈J , we use $A_{i,j}$ to denote its entry on the *i*-th row and *j*-th column, and use $A_{i,:}$ (or $A_{:,j}$ ) to denote its *i*-th row (or *j*-th column) vector. Let $\lambda_{k}\left(A\right)$ denote the *k*-th largest singular value of A for $k=1,\cdots ,\min_{\backslash lbra}ceN,J\backslash rbrace$ . Let $\left\|\cdot \right\|$ denote the spectral norm (operator norm) for matrices and $\ell_{2}$ norm for vectors, and ${\left\|\cdot \right\|}_{F}$ denote the Frobenius norm for matrices. For two non-negative sequences $\left\{a_{N}\right\}$ and $\left\{b_{N}\right\}$ , we write $a_{N}≲b_{N}$ (or $a_{N}≳b_{N}$ ) if there exists some constant C>0 independent of *N* such that $a_{N}\leq Cb_{N}$ (or $b_{N}\leq Ca_{N}$ ); $a_{N}≍b_{N}$ if $a_{N}≲b_{N}$ and $b_{N}≲a_{N}$ hold simultaneously; $a_{N}=o(b_{N})$ if $b_{N}>0$ and $a_{N}/b_{N}\to 0$ .

## 2 Latent class model with binary responses

We consider a dataset of *N* individuals (e.g., survey or assessment respondents and voters) and *J* items (e.g., questions in a survey or an assessment and policy items). The observed data are represented as a binary response matrix $R\in \left.l,b,r,a,ce0,1,r,b,r,a\right.ce^{N\times J}$ , where $R_{i,j}=1$ indicates a positive response from individual *i* to item *j*, and $R_{i,j}=0$ otherwise. Each individual *i* belongs to one of *K* latent classes, and we encode the class membership by a latent vector $s^{⋆}=\left(s_{1}^{⋆},\cdots ,s_{N}^{⋆}\right)$ , where $s_{i}^{⋆}\in K$ . The interpretation of the latent classes is characterized by an item parameter matrix $\Theta =(\theta_{j,k})\in {0,1}^{J\times K}$ , where $\theta_{j,k}$ denotes the probability that an individual in class *k* gives a positive response to item *j*:

(1) $$\begin{array}{c} P(R_{i,j}=1|s_{i}^{⋆}=k)=\theta_{j,k},\;\forall i\in N,j\in J. \end{array}$$

Under the LCM, a subject’s *J* responses are usually assumed to be conditionally independent given his or her latent class membership.

The traditional likelihood-based approach takes a random-effect perspective to LCMs and maximizes the marginal likelihood. This approach treats the latent class labels $\left\{s_{i}\right\}$ as random by assuming $p_{k}=P\left(s_{i}=k\right)$ for each *k* and marginalizing $s_{i}$ out:

(2) $$\begin{array}{c} L\left(\Theta |R\right)=\sum_{i\in N}\log\left(\sum_{k\in K}p_{k}\prod_{j\in J}\left(\theta_{j,k}^{R_{i,j}}{\left(1-\theta_{j,k}\right)}^{1-R_{i,j}}\right)\right). \end{array}$$

A standard approach for maximizing the marginal likelihood is the EM algorithm (Dempster et al., 1977). However, EM is highly sensitive to initialization. Poor initial values often lead to convergence at local maxima, so practitioners resort to multiple random restarts at substantial computational cost. Moreover, when both *J* and *K* grow large, the computation for evaluating the objective in (2) becomes numerically unstable due to its log-sum-of-products structure.

Another strategy is to consider the fixed-effect LCM, where the JML naturally comes into play. In particular, the joint log-likelihood function of latent label vector s and item parameters Θ can be written as

(3) $$\begin{array}{c} L\left(s,\Theta |R\right)=\sum_{i\in N}\sum_{j\in J}\left(R_{i,j}\log\left(\theta_{j,s_{i}}\right)+(1-R_{i,j})\log\left(1-\theta_{j,s_{i}}\right)\right). \end{array}$$

The simple structure of (3) implies that the optimization of JML can be decoupled across N and J . While JML is known to be inconsistent in low-dimensional settings (Neyman & Scott, 1948), the recent study by Zeng et al. (2023) has shown that JML can achieve consistent estimation of the latent labels in the high-dimensional regime. This property makes the JML approach appealing for large-scale modern datasets. It is worth noting that JML can be regarded as a version of classification EM (CEM; Celeux & Govaert, 1992), but with the difference of omitting the class proportion parameters. See Section 3.3 for more details.

Throughout the article, we consider the fixed-effect LCM and our goal is to recover the true latent labels $s^{⋆}$ from large-scale and high-dimensional data with N,J→∞ . The performance of any clustering method is evaluated via the Hamming error up to label permutations:

(4) $$\begin{array}{c} \ell \left(s,s^{⋆}\right):=\min_{\pi \in S_{K}}\frac{1}{N}\sum_{i\in N}I\left(s_{i}\neq \pi \left(s_{i}^{⋆}\right)\right), \end{array}$$

where $S_{K}$ denotes the set of all permutation maps π from K→K . This metric accounts for the inherent symmetry in clustering structure.

## 3 Two-stage clustering algorithm

### 3.1 Spectral clustering for the latent class model

Our primary goal is to estimate the latent label vector $s^{⋆}$ . However, maximizing the joint likelihood (3) directly still suffers from computationally intractable for large *N* and *J*. We first consider spectral clustering, which is a computationally efficient method that leverages the approximate low-rank structure of the data matrix. Let $Z=(Z_{i,j})\in \left.l,b,r,a,ce0,1,r,b,r,a\right.ce^{N\times K}$ be a latent class membership matrix with $Z_{i,k}=1$ if $s_{i}^{⋆}=k$ and $Z_{i,l}=0$ otherwise. Under the fixed-effect LCM, we have $R_{i,j}∼\text{Bern}\left(\sum_{k=1}^{K}Z_{i,k}\theta_{j,k}\right)$ and we can write $R_{i,j}=\sum_{k=1}^{K}Z_{i,k}\theta_{j,k}+E_{i,j}$ , where $\sum_{k=1}^{K}Z_{i,k}\theta_{j,k}=ER_{i,j}$ and $E_{i,j}$ is mean-zero random noise. We have the following crucial decomposition of the data matrix R into two additive components using matrix notation:

(5) $$\begin{array}{c} R=⏟Z_{N\times K}⏟{\Theta^{⊤}}_{K\times J}+⏟E_{\text{noise}}. \end{array}$$

Here, $Z\Theta^{⊤}=ER$ is the “signal” part containing the crucial latent class information (and thus is low-rank as $K\ll \min_{\backslash lbra}ceN,J\backslash rbrace$ ), and $E=(E_{i,j})\in R^{N\times J}$ is a mean-zero “noise” matrix with $EE=ER-ER]]=0_{N\times J}$ .

Given the above observation (5), we first briefly outline the rationale of spectral clustering under the oracle noiseless setting (i.e., when E=0 ). Let $Z\Theta^{⊤}=U\Sigma V^{⊤}$ be the rank-*K* SVD of $Z\Theta^{⊤}$ . Notably, the *i*th row vector in UΣ can be written as

(6) $$\begin{array}{c} e_{i}^{⊤}U\Sigma =e_{i}^{⊤}Z\Theta^{⊤}V=\sum_{k=1}^{K}Z_{i,k}\Theta_{:,k}^{⊤}V=\Theta_{:,s_{i}}^{⊤}V,\;\forall i\in N. \end{array}$$

**Figure 1:**
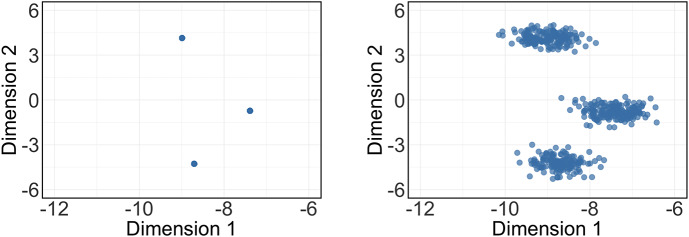
An illustration for spectral clustering: row vectors of UΣ (left) and $\hat{U}\hat{\Sigma }$ (right). Setting: N=500 , J=250 , and K=3 . Figure 1 long description.

Thus, data points in the same latent class have identical row vectors in UΣ , namely, one of the *K* distinct vectors $\left.l,b,r,a,ce\Theta_{:,k}^{⊤}V,r,b,r,a\right.ce_{k=1}^{K}$ . Hence, the true latent class label information can be readily read off from rows of UΣ . In the realistic noisy data setting, denote by $\hat{U}\hat{\Sigma }\hat{V^{⊤}}$ the top-*K* SVD of the data matrix R . Then intuitively, UΣ would be well-approximated by $\hat{U}\hat{\Sigma }$ provided that E is relatively small in magnitude, and the row vectors of $\hat{U}\hat{\Sigma }$ are noisy point clouds distributed around the *K* points. Therefore, K-means clustering can deliver reasonable estimates of class labels. We illustrate this point using an numerical example in Figure 1, where the row vectors of UΣ (left) and $\hat{U}\hat{\Sigma }$ (right) are plotted as points in $R^{2}$ , respectively.

The above high-level idea can be formalized as the spectral clustering algorithm (Algorithm 1), investigated in Zhang and Zhou (2024) for sub-Gaussian mixture models. Algorithm 1 first obtains the rank-*K* SVD of R denoted by $\hat{U}\hat{\Sigma }\hat{V^{⊤}}$ , and then performs K-means clustering on the rows of $\hat{U}\hat{\Sigma }$ , the left singular vectors weighted by the singular values.

Remark 1. An equivalent formulation of (3) aggregates the $2^{J}$ unique response patterns (weighted by their frequencies). We do not adopt this perspective because it becomes less suitable, both computationally and theoretically, for high-dimensional data with very large *J*. On the computational side, our approach does not rely on evaluating the likelihood through these unique response patterns. On the theoretical side, our spectral method is motivated by the approximate low-rank structure of the N×J binary response matrix $R\in R^{N\times J}$ itself, which does not utilize the unique response patterns.

**Algorithm 1:**
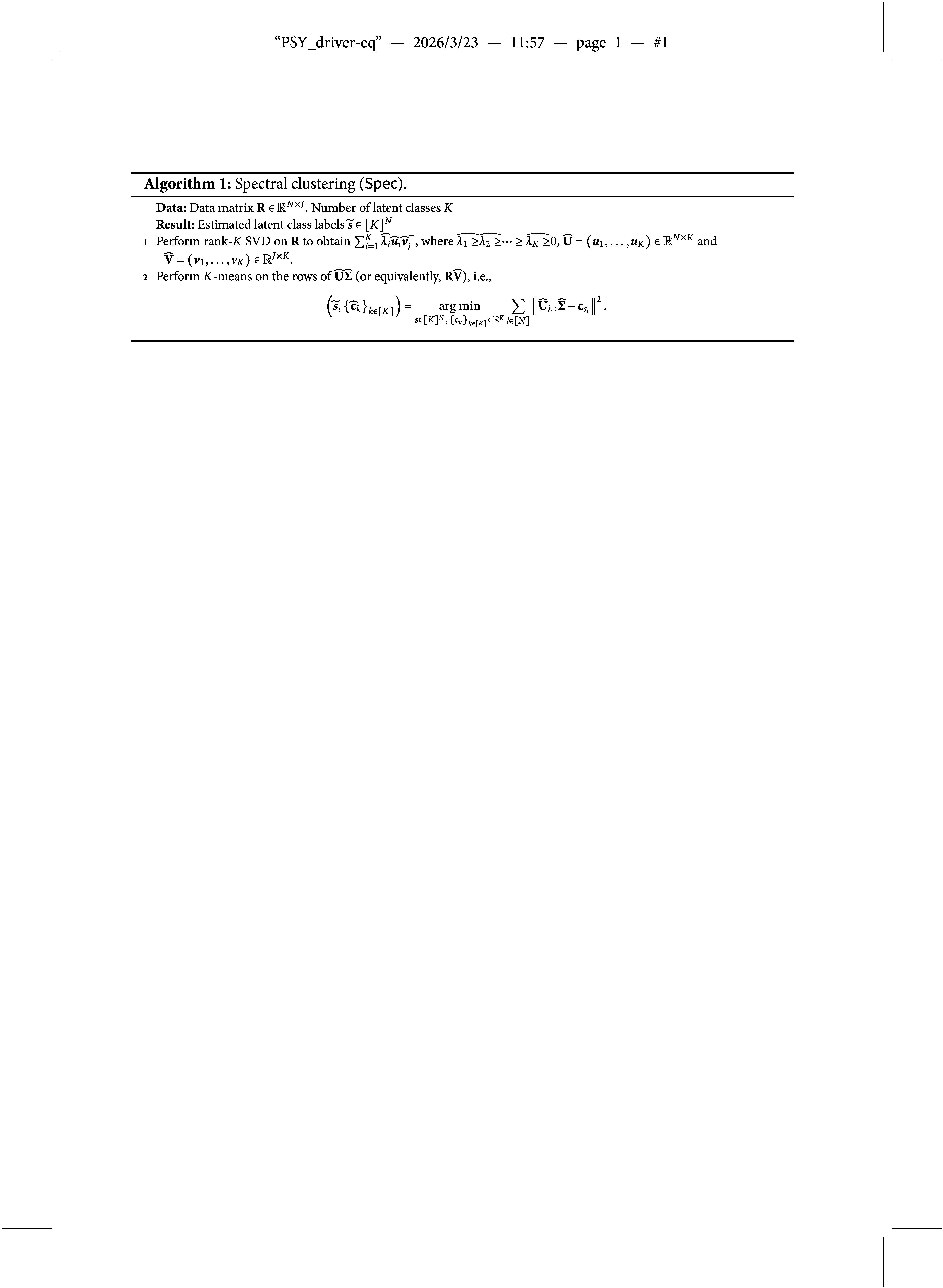
Algorithm 1 long description.

### 3.2 One-step likelihood refinement with spectral initialization

Despite computationally efficient, spectral clustering itself ignores the likelihood information in the LCM that might improve statistical accuracy. This motivates our proposed Algorithm 2 named spectral clustering with one-step likelihood refinement algorithm, or SOLA for short.

In particular, the first stage is to perform spectral clustering to obtain an initial class label estimate $\tilde{s}$ . This stage utilizes the inherent approximate low-rank structure of data but does not use the likelihood information. In the second stage, recall the joint likelihood function defined in (3) as

$$\begin{array}{c} L\left(s,\Theta |R\right)=\sum_{i\in N}\sum_{j\in J}\left(R_{i,j}\log\left(\theta_{j,s_{i}}\right)+(1-R_{i,j})\log\left(1-\theta_{j,s_{i}}\right)\right). \end{array}$$

We then perform the following two steps:

1. (Maximization) For fixed $\tilde{s}$ , find the Θ maximizing the objective $L\left(\tilde{s},\Theta |R\right)$ , which turns out to have a explicit solution as
$$\begin{array}{c} \hat{\theta_{j,k}}=\frac{\sum_{i\in N}R_{i,j}I\left(\tilde{s_{i}}=k\right)}{\sum_{i\in N}I\left(\tilde{s_{i}}=k\right)},\;\forall j\in J,\sim k\in K. \end{array}$$
  In other words, this is the sample average of the *j*-th column of R based on estimated class labels $\left.l,b,r,a,cei\in N:\tilde{s_{i}}=k,r,b,r,a\right.ce$ .
2. (Assignment) Given $\hat{\Theta }$ , the objective function $L(s,\hat{\Theta }|R)$ can be decoupled into *N* terms, each corresponding to an individual data point, and it is equivalent to assign label k∈K to sample *i* which maximize the log-likelihood independently for each i∈N :
$$\begin{array}{c} \hat{s_{i}}=\backslash operatorname*{\text{argmax}}_{k\in K}\sum_{j\in J}\left[R_{i,j}\log\left(\hat{\theta_{j,k}}\right)+(1-R_{i,j})\log\left(1-\hat{\theta_{j,k}}\right)\right],\;i\in N. \end{array}$$

It is readily seen that these two steps increase the joint likelihood monotonically. However, convergence to true $s^{⋆}$ depends on the performance of the initial estimator $\tilde{s}$ in the first stage, which we obtained using spectral clustering. The above details are summarized in Algorithm 2, and we provide its algorithmic guarantee in Section 4.

**Algorithm 2:**
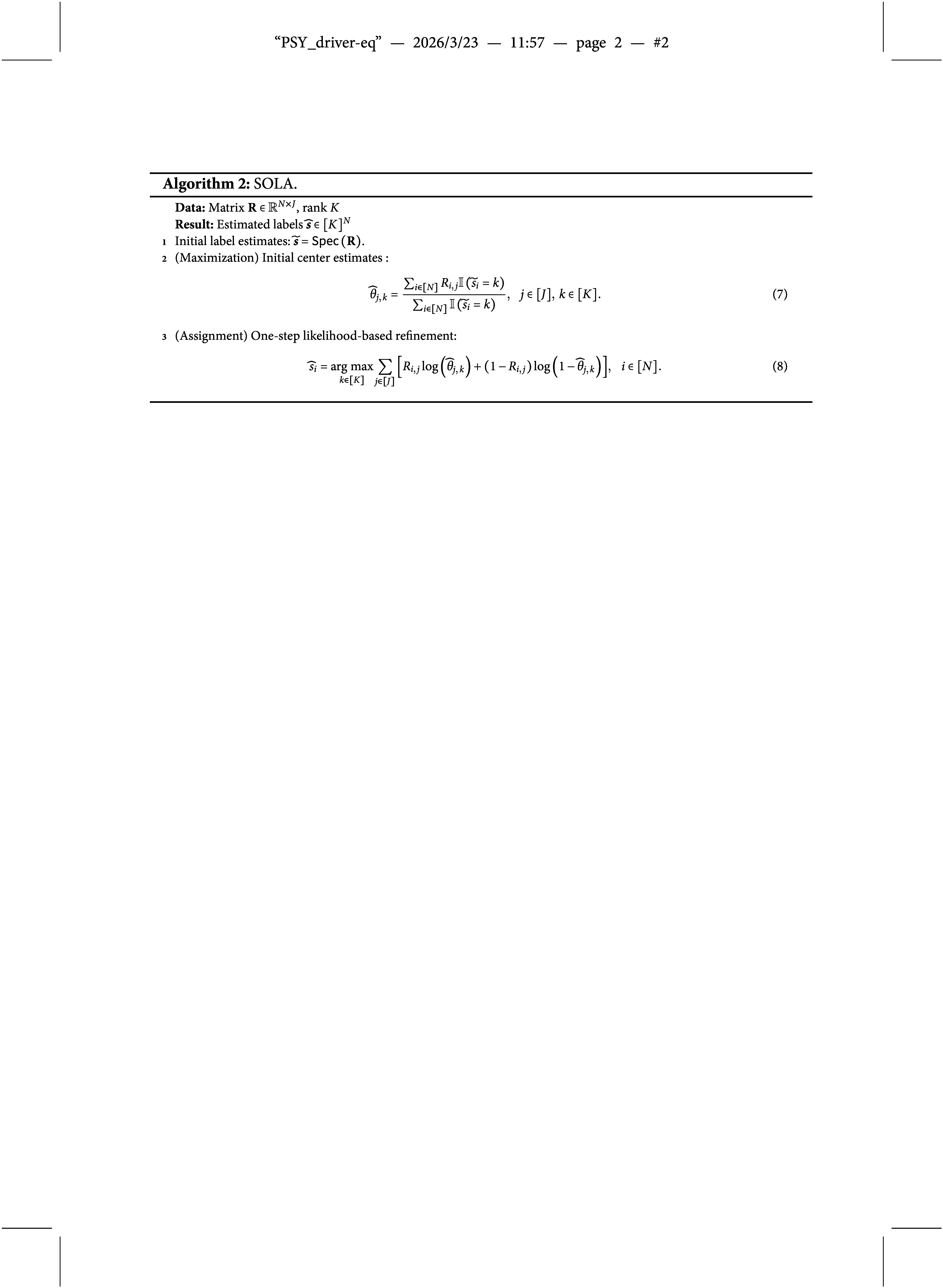
Algorithm 2 long description.

Interestingly, our two-stage procedure resonates with the spirit of the “initialize-and-refine” scheme that has been used in network analysis for stochastic block models (SBMs) (Chen et al., 2022; Gao et al., 2017; Lyu et al., 2023), while the model considered here differs fundamentally in that LCMs handle high-dimensional response data without relying on pairwise interaction structures inherent to SBMs.

In practice, we observe that performing multiple likelihood-based refinement steps may lead to improved convergence and performance compared to the one-step refinement procedure. Thus, we also consider a multiple-step refinement variant of Algorithm 2, which we denote by SOLA+, in the later simulation studies. We set the number of “Maximization + Assignment” refinement steps for SOLA+ to be 10 in all our simulations. In principle, one could also adapt the algorithm by monitoring the change in the joint log-likelihood values and stopping the algorithm once the absolute difference between successive iterations falls below a small threshold (Di Mari et al., 2023).

### 3.3 Extension: Classification EM refinement

Our proposed framework can be readily extended to one-step CEM refinement, where CEM is another widely used approach to estimate LCMs (Celeux & Govaert, 1992; Zeng et al., 2023). It can be regarded as a variant of the standard EM algorithm designed specifically for clustering. Unlike traditional EM, which calculates “soft” assignments by estimating the probability that each observation belongs to every latent class, CEM uses “hard” assignments: each observation is directly assigned to the class with the highest posterior probability. By explicitly modeling the mixture proportion parameters for the latent classes, CEM adjusts the likelihood to account for varying cluster sizes. Concretely, in our setting, one can adapt the one-step refinement in Algorithm 2 to a CEM step by including class proportions. Besides updating $\hat{\theta_{j,k}}$ as in (7), we also estimate the proportion parameters $\hat{p}=(\hat{p_{1}},\cdots ,\hat{p_{K}})\in {0,1}^{K}$ by

(9) $$\begin{array}{c} \hat{p_{k}}\;=\;\frac{1}{N}\sum_{i=1}^{N}I\{\tilde{s_{i}}=k\},\;k\in K. \end{array}$$

Then, for each individual *i*, we update their latent class label based on both the item parameters and the estimated class proportions:

(10) $$\begin{array}{c} \hat{s_{i}}\;=\;\backslash arg\max_{k\in K}\left[\log\hat{p_{k}}\;+\;\sum_{j=1}^{J}\left(R_{i,j}\log\hat{\theta_{j,k}}\;+\;(1-R_{i,j})\log\left(1-\hat{\theta_{j,k}}\right)\right)\right]. \end{array}$$

This one-step CEM update replaces the likelihood-refinement stage of our two-stage estimator: start with spectral clustering, then perform the CEM step above. We provide its theoretical guarantee in Section 4. In practice, one could easily generalize the above CEM procedure to a multiple-step refinement variant, which is analogous to SOLA+.

## 4 Theoretical guarantees

To deliver the theoretical results, we start by making the following mild model assumptions.

Assumption 1.
$\theta_{j,k}\in c_{\theta },C_{\theta }$ for some universal constants $0<c_{\theta }<C_{\theta }<1$ , and j∈J , k∈K .

Assumption 2.
$\min_{k\in K}\left|\left.l,b,r,a,cei\in N:s_{i}^{⋆}=k,r,b,r,a\right.ce\right|\geq \alpha N/K$ for some universal constant α∈(0,1] .

Assumption 1 is a standard assumption for considering a compact parameter space for Θ . Assumption 2 is imposed for both practical consideration and technical reason. Without this, rare classes could be statistically indistinguishable and computationally unstable to estimate.

### 4.1 Theoretical guarantee of spectral clustering

In this section, we introduce the theoretical guarantee of spectral clustering (Algorithm 1). To that end, we define some necessary notations. The difficulty of recovering $s^{⋆}$ depends on how distinct the latent classes are, which, in the context of spectral clustering, is characterized by the following quantity:

(11) $$\begin{array}{c} \Delta :=\min_{k_{1}\neq k_{2}\in K}\left\|\Theta_{:,k_{1}}-\Theta_{:,k_{2}}\right\|=\min_{k_{1}\neq k_{2}\in K}{\left[\sum_{j=1}^{J}{\left(\theta_{j,k_{1}}-\theta_{j,k_{2}}\right)}^{2}\right]}^{1/2}. \end{array}$$

This quantity measures the minimal Euclidean distance between any two class-specific item parameter vectors. Intuitively, a larger Δ yields a larger gap between the columns of Θ and a bigger distinction among the latent classes, and therefore produces clearer separation in the spectral embeddings (referring to (6)), making recovery of the true class labels easier. In addition, let $\lambda_{1}\geq \cdots \geq \lambda_{K}>0$ denote the singular values of $ER=Z\Theta^{⊤}$ in decreasing order. The following result characterizes the clustering error of Algorithm 1.

Proposition 1 **(Adapted from Theorem 3.1 in Zhang & Zhou, 2024).**

Define $\sigma_{\theta }^{2}:=\left(1-2\theta \right)/\left[2\log\left(\left(1-\theta \right)\theta^{-1}\right)\right]$ for $\theta \in (0,1)\backslash \left.l,b,r,a,ce1/2,r,b,r,a\right.ce$ and $\sigma_{\theta }^{2}:=1/4$ for θ=1/2 , and let $\bar{\sigma }:=\max_{j,k}\sigma_{\theta_{j,k}}$ . Assume $\text{rank}\left(\Theta \right)=K$ , $\alpha N/K^{2}\geq 10$ and

(12) $$\begin{array}{c} \min_{\backslash lbra}ce\frac{\Delta }{\alpha^{-1/2}K\left(1+\sqrt{\frac{J}{N}}\right)\bar{\sigma }},\frac{\lambda_{K}}{\left(\sqrt{N}+\sqrt{J}\right)\bar{\sigma }}\backslash rbrace\to \infty . \end{array}$$

Then, for the spectral clustering estimator $\tilde{s}$ in Algorithm 1, we have

(13) Eℓs~,s⋆≤\exp−Δ28^¯σ21−o(1)+\exp−N2,

where ℓ(⋅,⋅) is defined in (4). Moreover, if further assume that Δ2≥81+c^¯σ2logN for any constant c>0 , then we have $\ell \left(\tilde{s},s^{⋆}\right)=0$ with probability $1-\backslash exp\left(-N/2\right)-\backslash exp\left(-\Delta /\bar{\sigma }\right)$ .

Proposition 1 delivers that the simple and computationally efficient spectral clustering can essentially lead to exponentially small error rate with respect to the latent class separation Δ . Notably, we can have exact recovery of all latent class labels (i.e., $\ell \left(\tilde{s},s^{⋆}\right)$ with $\tilde{s_{i}}=\pi (s_{i})$ for all i=1,⋯,N ) with high probability as long as Δ2/^¯σ2≥81+clogN . In the LCM we study, we have $\bar{\sigma }\leq 1/4$ by definition, and hence it suffices to have $\Delta^{2}\geq 2(1+c)\log N$ to achieve exact recovery. This is not hard to achieve even for slowly growing number of items, because if we have constant separation of item parameters for each item with $\delta =\min_{k_{1}\neq k_{2}\in K,j\in J}|\theta_{j,k_{1}}-\theta_{j,k_{2}}|$ , then $\Delta^{2}=J\delta^{2}$ . In this case, having $J>2(1+c)\delta^{-2}\log N$ for exact recovery corresponds to a moderately growing dimension. In sharp contrast, the JML estimator analyzed in Zeng et al. (2023) only achieves a polynomial convergence rate of order $O(J^{-1/2})$ . Moreover, they cannot achieve exact recovery according to the technical condition and conclusion therein. Therefore, the new spectral estimator $\tilde{s}$ represents a substantial improvement with exponential error decay in (13).

We also note that the condition (12) on Δ and $\lambda_{K}$ is mild and can be easily satisfied in the modern high-dimensional data setting. We next give a concrete example. Let us consider a common generative setting for item parameters Θ , where its entries are i.i.d. generated from the Beta distribution.

Proposition 2. Consider $\theta_{j,k}\backslash stackreli.i.d.∼\text{Beta}(a,b)$ for all j∈J and k∈K with a,b>0 . Define

$$\begin{array}{c} B:=\frac{a\left(a+b+a{\left(a+b\right)}^{-1}\right)}{\left(a+b\right)\left(a+b+1\right)}, \end{array}$$

then, under Assumption 2, we have $\lambda_{K}≳\sqrt{\alpha BNJ}$ and $\Delta ≳\sqrt{BJK}$ with probability at least $1-K(e^{-c_{1}B^{2}J}+e^{-c_{2}BJ})$ for some universal constants $c_{1},c_{2}\in (0,1)$ .

Proposition 2 entails that the condition on Δ and $\lambda_{K}$ for the success of spectral clustering in Proposition 1 can be easily satisfied when Θ consists of Beta distributed entries, a common assumption in Bayesian inference for the LCM. Specifically, combined with Assumption 1, a sufficient condition on *B* for spectral clustering to have the desirable error control (13) would be $B\gg K(\frac{1}{N}+\frac{1}{J})$ , which is easily satisfied if the Beta parameters *a* and *b* are of the constant order.

Despite the above nice theoretical guarantee for spectral clustering, the clustering error’s reliance on the Euclidean metric Δ in Proposition 1 is inherent from the general framework analyzed in Zhang and Zhou (2024), and it turns out to be not statistically optimal due to ignorance of the likelihood information. We will discuss and address this issue next.

### 4.2 Fundamental statistical limit for LCMs

In this section, we provide the understanding of the information-theoretic limit (fundamental statistical limit) of clustering in LCMs. To compare different statistical procedures, we consider the minimax framework that had been widely considered in both statistical decision theory and information theory in the past decades (Le Cam, 2012; Takezawa, 2005). The minimax risk is defined as the infimum, over all possible estimators, of the maximum loss (here, the mis-clustering error) taken over the entire parameter space. In other words, it is the rate of the best estimator one can have among all statistical procedures in the worst-case scenario.

Formally, we establish the minimax lower bound for mis-clustering error under the fixed-effect LCM in the following result, whose proof is adapted from Lyu et al. (2025) and can be found in Section B.1.

Theorem 1 **(Minimax lower bound).**

Consider the following parameter space for ER :

$$\begin{array}{cc} & P_{K}\left(s,\Theta \right):=\left.l,b,r,a,ce\bar{R}:\bar{\_}Ri,j=\theta_{j,s_{i}},s\in K^{N},\theta_{j,k}\in 0,1,\forall i\in N,j\in J,k\in K,r,b,r,a\right.ce. \end{array}$$

Define $I^{⋆}:=\min_{k_{1}\neq k_{2}\in K}\sum_{j\in J}I(\theta_{j,k_{1}},\theta_{j,k_{2}})$ with

$$\begin{array}{c} I(p,q):=-2\log\left(\sqrt{pq}+\sqrt{\left(1-p\right)\left(1-q\right)}\right),\;\forall p,q\in (0,1), \end{array}$$

then we have

(14) $$\begin{array}{c} \underset{\hat{s}}{\inf}\underset{P_{K}\left(s,\Theta \right)}{\sup}E\ell \left(\hat{s},s^{⋆}\right)\geq \backslash exp\left(-\frac{I^{⋆}}{2}\left(1+o(1)\right)\right), \end{array}$$

where the infimum is taken over all possible estimators of the latent class labels.

Roughly speaking, Theorem 1 informs us that under the fixed-effect LCM, the error rate $\backslash exp(-I^{⋆}/2)$ is the lowest possible clustering error rate that no estimator can uniformly surpass. Notably, I(p,q) is exactly the Renyi divergence (Rényi, 1961) of order 1/2 between two Bernoulli distributions, Bern (p) and Bern (q) , and hence $\sum_{j\in J}I(\theta_{j,k_{1}},\theta_{j,k_{2}})$ can be interpreted as the Renyi divergence between two Bernoulli random vectors with parameters $\Theta_{:,k_{1}}$ and $\Theta_{:,k_{2}}$ , respectively. $I^{⋆}$ can be regarded as the overall signal-to-noise ratio (SNR) which further takes account into all possible pairs of $(k_{1},k_{2})\in K^{2}$ with $k_{1}\neq k_{2}$ . Although the form of $I^{⋆}$ might seem complicated at its first glance, it actually exactly reflects the likelihood information in the LCMs. By definition of the minimax lower bound, we know that $\backslash exp(-I^{⋆}/2)$ cannot be larger than the spectral clustering error rate \exp(−Δ2/(8^¯σ2)) obtained in Proposition 1. We will discuss this in more detail in Section 4.4.

In the following, we take a closer look at why the quantity $I^{⋆}$ shows up as the fundamental statistical limit. We analyze an idealized scenario where the true item parameters Θ are known, which we term as the *oracle* setting. For simplicity, we consider K=2 latent classes and $I^{⋆}=\sum_{j\in J}I(\theta_{j,1},\theta_{j,2})$ . In this regime, we can relate the clustering problem for the *i*-th sample to the following binary hypothesis testing problem:

$$\begin{array}{c} H_{0}:s_{i}^{⋆}=1,\;\text{v.s.}\;H_{0}:s_{i}^{⋆}=2. \end{array}$$

By the Neyman–Pearson lemma, the test that gives the optimal Type-I plus Type-II error is the likelihood ratio test. Consequently, the oracle classifier assigns an observation to the class that maximizes the log-likelihood:

(15) $$\begin{array}{c} \bar{\_}si=\backslash operatorname*{\text{argmax}}_{k\in 2}\sum_{j\in J}\left[R_{i,j}\log\theta_{j,k}+(1-R_{i,j})\log\left(1-\theta_{j,k}\right)\right]. \end{array}$$

Assuming without loss of generality that $s_{i}^{⋆}=1$ , one can show that the mis-clustering probability satisfies

$$\begin{array}{cc} & P\left(\bar{\_}si\neq s_{i}^{⋆}\right)=P\left(\sum_{j\in J}R_{i,j}\log\frac{\theta_{j,2}\left(1-\theta_{j,1}\right)}{\theta_{j,1}\left(1-\theta_{j,2}\right)}\geq \sum_{j\in J}\log\frac{1-\theta_{j,1}}{1-\theta_{j,2}}\right)\leq \backslash exp\left(-\frac{\sum_{j\in J}I(\theta_{j,1},\theta_{j,2})}{2}\right). \end{array}$$

In other words, the above error rate in the oracle setting directly informs the information-theoretic limit of any clustering procedure in the LCM with binary responses, as indicated by Theorem 1. Note that the oracle estimator (15) that achieves this optimal rate is constructed using the true Θ and cannot be applied in practice. Later, we will construct a computationally feasible estimator (i.e., our SOLA estimator) that achieves this optimal rate without relying on prior knowledge of Θ .

### 4.3 Theoretical guarantee of SOLA

In this section, we give the theoretical guarantee of SOLA. To facilitate the theoretical analysis, we consider a sample-splitting variant of SOLA (Algorithm 3), where the sample data points are randomly partitioned into two subsets $S_{1},S_{2}$ of size ⌈N/2⌉ and N−⌈N/2⌉ . The key idea is to estimate Θ using one subset of samples, and then update labels for the remaining ones. The procedure can be repeated by switching the roles of the two subsets, and we can obtain labels for all samples. Finally, an alignment step is needed due to the permutation ambiguity of clustering in the two subsets. We have the following result, whose proof is deferred to Section B.2.

**Algorithm 3:**
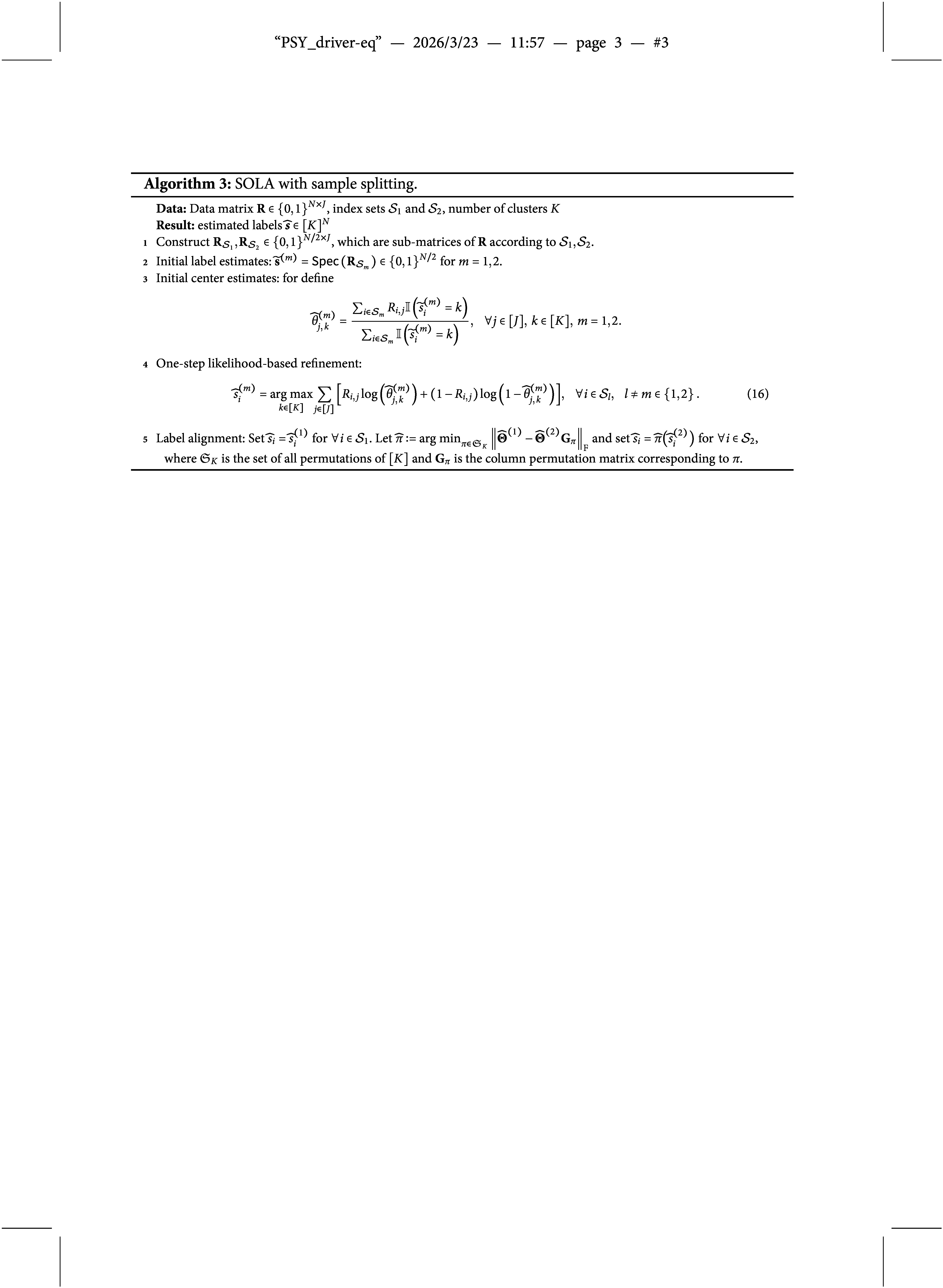
Algorithm 3 long description.

Theorem 2. Suppose Assumptions 1 and 2 and the assumptions of Proposition 1 hold. In addition, assume that N≳KlogN , JK≲min\exp(−cΔ2/^¯σ2),Nγ0} , for some universal constants 0<c<1/8 and $\gamma_{0}>0$ . If the SNR condition satisfies

$$\begin{array}{c} \frac{\Delta /\bar{\sigma }}{\sqrt{J}K^{3/4}{\left(\log N\right)}^{1/4}/N^{1/4}}\to \infty , \end{array}$$

then, for $\hat{s}$ from Algorithm 3, we have

$$\begin{array}{c} E\ell \left(\hat{s},s^{⋆}\right)\leq \backslash exp\left(-\frac{I^{⋆}}{2}\left(1-o(1)\right)\right). \end{array}$$

Combining Theorems 1 and 2, we can see that the estimator of Algorithm 3 achieves the fundamental statistical limit and is optimal for estimating the latent class labels. We have several remarks on Theorem 2. First, it is interesting to consider the regime when $J=o(\sqrt{\frac{N\log N}{K}})$ , since otherwise the SNR condition would already implies exact recovery (perfect estimation of latent class labels with high probability) for spectral clustering, as shown by Theorem 2 and Proposition 1. Second, the sample splitting step is crucial in the current proof of Theorem 2 to decouple the dependence between $R_{i,j}$ and $\hat{\Theta }$ in (8). This step serves purely as a theoretical device to facilitate the theoretical analysis of clustering error rate, as commonly adopted in theoretical statistics (Abbe, 2018; Lei & Zhu, 2017; Rinaldo et al., 2019; Vu, 2018). In practice, halving the sample only increases the estimation error of $\hat{\Theta }$ by a constant factor, which is negligible in theory and immaterial in practice when the sample size *N* is large. Alternatively, it may be possible in the future to show that the same theoretical guarantee holds for the original version of SOLA, that is, Algorithm 2, by developing a leave-one-out analysis similar to that in Zhang and Zhou (2024). However, the analysis will be highly technical and much more involved than that in Zhang and Zhou (2024) and beyond the scope of our current focus. Last, although sample splitting simplifies theoretical analysis, it may not be necessary in practice; we use the original SOLA (Algorithm 2) for all numerical experiments and observe satisfactory empirical results.

Remark 2. Our work is related to the Tensor-EM method introduced by Zeng et al. (2023), which also follows an initialization-and-refinement strategy. In the high level, both our methods require moment-based methods in the initialization. However, there are two important distinctions. Zeng et al. (2023) employ a tensor decomposition that leverages higher-order moments and can be computationally intensive and unstable. In contrast, our spectral initialization exploits only the second moment of data (since SVD of the data matrix is equivalent to eigendecomposition of the Gram matrix), making it both simple and efficient. Second, the error rate established in Zeng et al. (2023) is of polynomial order, that is, $O\left(J^{-1/2}\right)$ , whereas our SOLA achieves the statistically optimal exponential error rate with respect to $I^{⋆}$ .

As an extension of Theorem 2, we have a similar theoretical guarantee for the one-step CEM refinement proposed in Section 3.3, whose proof is given in Section B.4.

Corollary 1. Suppose the conditions in Corollary 2 hold. Consider the output of Algorithm 3 with (16) replaced by (9) and sample splitting version of (10), we have

$$\begin{array}{c} E\ell \left(\hat{s},s^{⋆}\right)\leq \backslash exp\left(-\frac{I^{⋆}}{2}\left(1-o(1)\right)\right). \end{array}$$

Theorem 1 indicates that one-step CEM refinement achieves the same exponential error rate $\backslash exp(-I^{*}/2)$ as Algorithm 3. There might be a gap between these two methods when Assumption 2 does not hold, which is an interesting future direction to consider.

### 4.4 Comparison of spectral clustering and SOLA

In this section, we compare the convergence rate of spectral clustering and SOLA under LCM. While spectral clustering is widely employed due to its computational efficiency and solid theoretical guarantees in general clustering settings, we demonstrate that SOLA attains minimax-optimal clustering error rates in LCMs that spectral clustering fails to achieve.

In view of Theorem 1, the minimax-optimal clustering error rate for LCM is of order $\backslash exp(-I^{⋆}/2)$ . On the other hand, spectral clustering is shown to achieve the error rate scaling as \exp(−Δ2/(8^¯σ2)) in Proposition 1. To understand the difference between these two quantities, we have the following result, whose proof can be found in Section B.5.

Proposition 3. Assume that (a) $\min_{k_{1}\neq k_{2}\in K}\left|\Omega_{0}(k_{1},k_{2})\right|≳J$ , where $\Omega_{0}(k_{1},k_{2}):=\{j\in J:\left|\theta_{j,k_{1}}-\theta_{j,k_{2}}\right|=o(1)\}$ and (b) $\min_{k\in K}\left|\left.l,b,r,a,cej\in J:\left|\theta_{j,k}-1/2\right|>c_{0},r,b,r,a\right.ce\right|≳J$ for some universal constant $c_{0}>0$ . Then we have

\exp−I⋆2≲\exp−Δ28^¯σ21+c,

for some universal constant c>0 .

A few comments are in order regarding Proposition 3. First, under the conditions (a) and (b), the rate of spectral clustering becomes sub-optimal (in order) due to a loose constant in the exponent as the latent class separation Δ→∞ . Second, it is important to note that the mis-clustering error $\ell (\hat{s},s^{*})$ takes values in the finite set $\left.l,b,r,a,ce0,1/N,\cdots ,1,r,b,r,a\right.ce$ according to the definition (4). Given this, it is only meaningful to compare error rates when the exponent Δ2/^¯σ2 (or $I^{⋆}$ ) does not exceed a constant factor of logN , since otherwise exact clustering of all subjects is achieved with high probability. To see this, we use Markov inequality to get

$$\begin{array}{c} P(\ell (\hat{s},s^{*})\neq 0)=P(\ell (\hat{s},s^{*})\geq 1/N)\leq NE\ell (\hat{s},s^{*})\leq N\backslash exp\left(-C\log N\right)\to 0, \end{array}$$

for a constant C>1 . In view of this, the condition (a) helps us exclude the trivial case where $I^{⋆}≍\Delta^{2}≍J$ , which would only allow J≲logN . Third, the essential order difference occurs when most of $\theta_{j,k}$ ’s are bounded away from 1/2 . To see this, consider the scenario when K=2 , $\theta_{j,1}=0.5$ , and $\theta_{j,2}=0.5-\delta$ for some δ=o(1) . Then we have $\Delta =\frac{1}{2}J\delta^{2}$ and $I^{⋆}=-J\log\left(\sqrt{\frac{1}{2}\left(\frac{1}{2}-\delta \right)}+\sqrt{\frac{1}{2}\left(\frac{1}{2}+\delta \right)}\right)=\frac{1}{2}J\delta^{2}\left(1+o(1)\right)$ . This implies that without condition (b), the exponents Δ and $I^{⋆}$ are of the same order, resulting in at most a constant-factor difference in clustering error for spectral clustering and SOLA. We also conduct numerical experiments in Section 5 to verify the necessity of (b).

### 4.5 Estimation of the number of latent classes *K*

In practice, the true number of latent classes *K* may be unknown. To see how we can estimate *K* from data, recall that $R=Z\Theta^{⊤}+E$ . By Weyl’s inequality and classical random-matrix theory (Vershynin, 2018), the singular values of R satisfy

$$\begin{array}{c} \left|\lambda_{i}\left(R\right)-\lambda_{i}\left(Z\Theta^{⊤}\right)\right|\leq \left\|E\right\|=O_{p}\left(\sqrt{J}+\sqrt{N}\right),\;i\in N\wedge J. \end{array}$$

Since $Z\Theta^{⊤}$ has exactly *K* nonzero singular values $\lambda_{1}\geq \cdots \geq \lambda_{K}>0$ , and Proposition 2 shows that $\lambda_{K}≳\sqrt{\alpha BNJ}$ under a common generative setting, the top *K* singular values of R lie well above the noise level. Accordingly, we estimate *K* by counting how many singular values of R exceed a threshold slightly above the noise level:

(17) $$\begin{array}{c} \hat{K}=\left|\left.l,b,r,a,cei\in N\wedge J:\lambda_{i}\left(R\right)>2.01\left(\sqrt{J}+\sqrt{N}\right),r,b,r,a\right.ce\right|. \end{array}$$

Here, the factor 2.01 ensures we count only those singular values contributed by the low-rank signal. We have the following lemma certifying the consistency of $\hat{K}$ , whose proof is deferred to Section B.6.

Lemma 1. Instate the assumptions of Proposition 1. For $\hat{K}$ defined in (17), we have

$$\begin{array}{c} P\left(\hat{K}=K\right)=1-o(1),\;\text{as\textasciitilde{}}N,J\to \infty . \end{array}$$

This thresholding rule provides a formal analog of the classical scree-plot method (Cattell, 1966; Zhang et al., 2020), and its proof shares a similar spirit of Theorem 2 in Zhang et al. (2020) but with a sharper characterization of the constant factor in the noise magnitude.

## 5 Numerical experiments

### 5.1 Simulation studies

We compare our proposed methods SOLA (Algorithm 2) and SOLA+ (Algorithm 2 with multiple steps refinement), with spectral method (Zhang & Zhou, 2024), EM (Linzer & Lewis, 2011), and Tensor-CEM (Zeng et al., 2023). For the EM algorithm, we employ the popular polca package (Linzer & Lewis, 2011) in R, and for Tensor-CEM, we use the original MATLAB code (Zeng et al., 2023) for tensor initialization combined with our R-based implementation of the CEM refinement. Our evaluation focuses on three aspects: (a) clustering accuracy, (b) stability, and (c) computational efficiency. Throughout the simulations, we generate the true latent label $\left.l,b,r,a,ces_{i}^{⋆},r,b,r,a\right.ce_{i=1}^{N}$ independently uniformly from K and set K=3 , N=2J . The parameters $\theta_{j,k}$ are independently sampled from Beta $\left(\beta_{1},\beta_{2}\right)$ . We generate 200 independent replicates and report the average mis-clustering error and computation time, excluding any replicates in which the algorithm fails (Tensor-CEM and EM). Notably, our algorithm is numerically stable without any failures in all simulations.

Remark 3 **(EM implementations: polca vs. LatentGOLD).**

Beyond polca, we also evaluated LatentGOLD (Vermunt & Magidson, 2005), which implements a robust EM routine with safeguards against numerical underflow. Table 1 reports a representative comparison across three regimes ( N=2J , N=J , and N=J/2 ), showing that LatentGOLD is substantially more stable and accurate than polca in these settings. Our overall message remains unchanged: EM-type methods can be competitive in the classical regime with relatively large *N* and smaller *J*, whereas SOLA/SOLA+ become increasingly advantageous as *J* grows and the problem enters the high-dimensional regime (with *J* comparable to or larger than *N*). While retaining polca for comparison purposes in our large-scale simulation studies, we emphasize that when analyzing data with a small-to-moderate *J*, it is recommended to use LatentGOLD for their better numerical stability and performance.

**Table 1:** Clustering error across different methods and settings under 100 replicates Table 1 long description.

Setting/Method	SOLA	SOLA+	EM by polca	EM by LatentGold
N=190,J=95 ( N=2J )	0.0815	0.0772	0.3022	0.0645
N=95,J=95 ( N=J )	0.1760	0.1745	0.4642	0.2609
N=75,J=150 ( N=J/2 )	0.0411	0.0411	0.5002	0.2795

**Figure 2:**
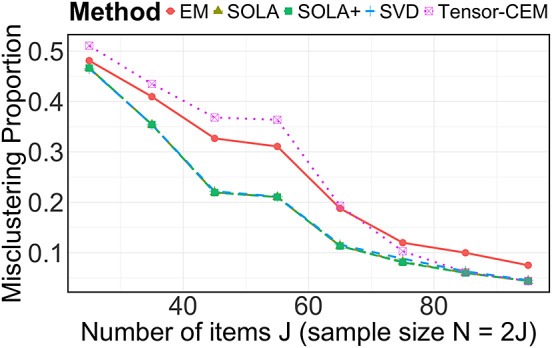
Simulation 1: Mis-clustering proportions versus number of items *J*. Entries of Θ are independently generated from Beta(5,5) . Figure 2 long description.

*Simulation 1: Latent class estimation accuracy for dense* Θ *close to* 1/2 . In the first simulation, we set $\left(\beta_{1},\beta_{2})=(5,5\right)$ . Under these parameters, the entries of Θ tend to be close to 0.5 . As shown in Figure 2, our proposed methods achieve lower mis-clustering errors compared to the alternative approaches. In this setting, the improvement of SOLA over the plain spectral clustering method is negligible, which is expected since $\theta_{j,k}$ ’s values near 0.5 reduce the additional gain from incorporating the likelihood information. This observation supports the necessity of having $\theta_{j,k}$ ’s well separated from 0.5 (see Proposition A.1) to have likelihood-based refinement improve upon spectral clustering. It is also worth noting that Tensor-CEM performs poorly when both the number of items *J* and the sample size *N* are small, likely due to instability introduced by its tensor-based initialization step.

**Figure 3:**
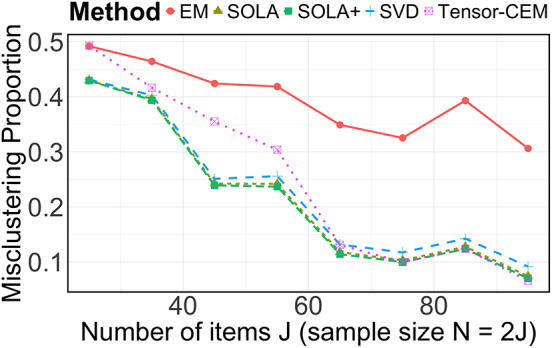
Simulation 2: Mis-clustering proportions versus number of items *J*. Entries of Θ are independently generated from Beta(1,8) . Figure 3 long description.

*Simulation 2: Latent class estimation accuracy for sparse item parameters* Θ . In the second simulation, we choose $\left(\beta_{1},\beta_{2})=(1,8\right)$ , a sparse scenario, where the $\theta_{j,k}$ ’s tend to be close to 0 . Figure 3 illustrates that our proposed methods exhibit superior clustering accuracy when *N* and *J* are small, while maintaining competitive performance as these dimensions increase. In this sparse setting, the traditional EM algorithm performs the worst because $\theta_{j,k}$ ’s are near the boundary (close to 0 ), making it difficult for random initializations to converge to optimal solutions. Furthermore, the gap between spectral clustering and SOLA becomes more pronounced when the $\theta_{j,k}$ ’s are bounded away from 0.5 .

**Figure 4:**
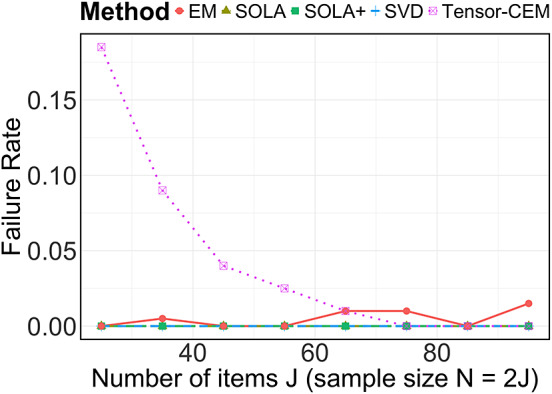
Simulation 3: Failure rate versus the number of items *J*. Entries of Θ are independently generated from Beta(1,8) . Figure 4 long description.

*Simulation 3: Stability analysis.* We next assess the stability of the different methods under the sparse setting $\left(\beta_{1},\beta_{2})=(1,8\right)$ . In particular, we record a “failure” point if either (a) the estimated number of classes degenerates from the pre-specified *K* to a smaller value or (b) the algorithm produces an error during execution. As shown in Figure 4, our proposed methods are robust across a wide range of *N* and *J* without any failures in any simulation trial. In particular, the failure rate for Tensor-CEM exceeds 15% for small *N* and *J*, likely due to its sensitive initialization, while the EM algorithm becomes increasingly unstable as the dimensions grow. These results underscore the robustness of our SOLA approaches.

**Figure 5:**
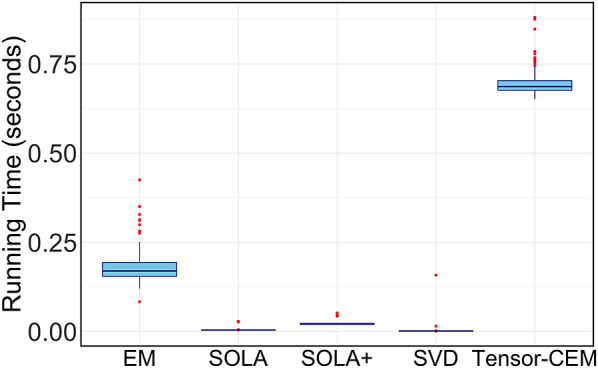
Simulation 4: Running time (seconds) of different methods. Figure 5 long description.

*Simulation 4: Computational efficiency.* Finally, we compare the computation time required by each method. In our experiments, the SVD step and our SOLA and SOLA+ algorithms are implemented in R. The total computation time for Tensor-CEM is the sum of the MATLAB initialization and the R refinement stages. As reported in Table 2 and illustrated in Figure 5, our proposed methods are significantly faster than both the EM and Tensor-CEM approaches, with computation time comparable to that of SVD.

**Table 2:** Simulation 4: Running time (seconds) of different methods Table 2 long description.

	SVD	SOLA	SOLA+	EM	Tensor-CEM
J=25	$4.15\times 10^{-4}$	$8.62\times 10^{-4}$	$4.19\times 10^{-3}$	$1.42\times 10^{-2}$	$5.12\times 10^{-1}$
J=95	$2.33\times 10^{-3}$	$4.66\times 10^{-3}$	$2.51\times 10^{-2}$	$1.80\times 10^{-1}$	$6.95\times 10^{-1}$

**Figure 6:**
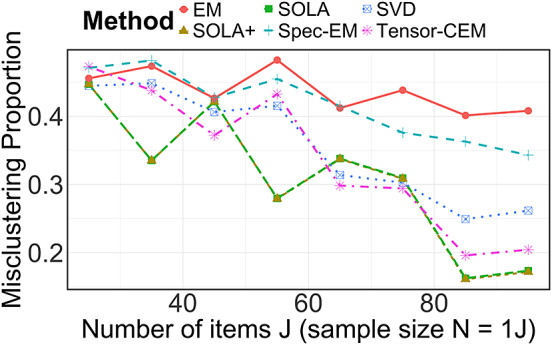
Simulation 5-1: Mis-clustering proportions versus number of items *J* under N=J . Entries of Θ are independently generated from Beta(1,8) . Figure 6 long description.

**Figure 7:**
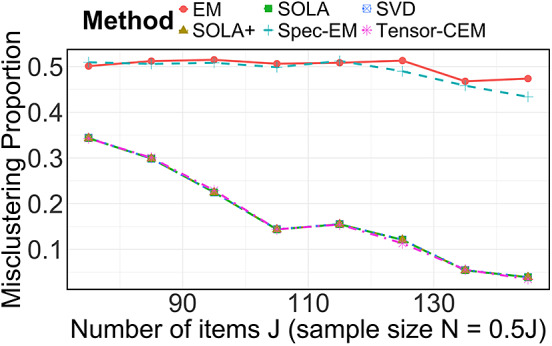
Simulation 5-2: Mis-clustering proportions versus number of items *J* under N=0.5J . Entries of Θ are independently generated from Beta(1,8) . Figure 7 long description.

**Figure 8:**
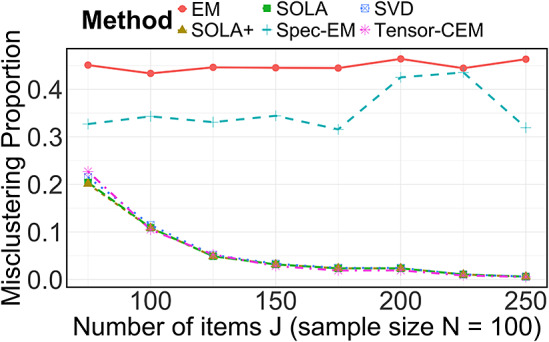
Simulation 5-3: Mis-clustering proportions versus number of items *J* under N=100 . Entries of Θ are independently generated from Beta(1,8) . Figure 8 long description.

**Figure 9:**
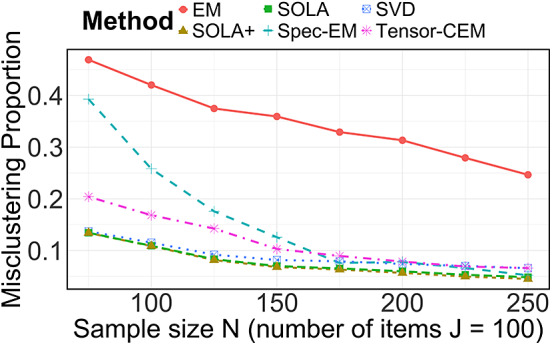
Simulation 5-4: Mis-clustering proportions versus sample size *N* under J=100 . Entries of Θ are independently generated from Beta(1,8) . Figure 9 long description.

**Figure 10:**
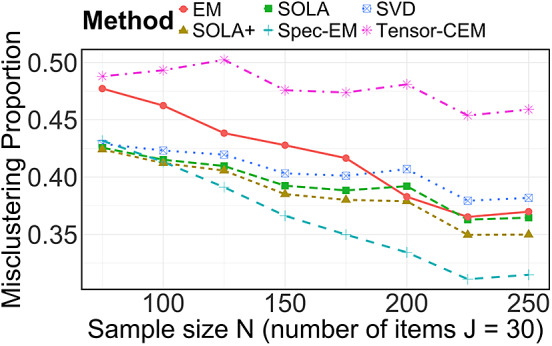
Simulation 5-5: Mis-clustering proportions versus sample size *N* under J=30 . Entries of Θ are independently generated from Beta(1,8) . Figure 10 long description.

**Figure 11:**
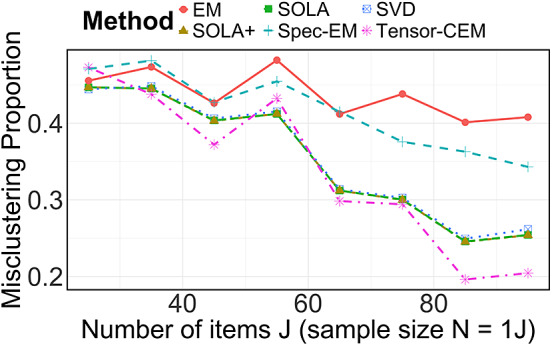
Simulation 5-6: Mis-clustering proportions versus number of items *J* under imbalanced latent classes with N=J . Entries of Θ are independently generated from Beta(1,8) . Figure 11 long description.

**Figure 12:**
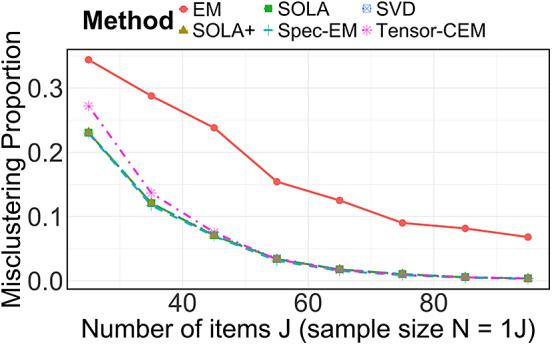
Simulation 5-7: Mis-clustering proportions versus number of items *J* under N=J . The Θ matrix is set to a fixed design matrix. Figure 12 long description.

*Simulation 5: Latent class estimation accuracy under different regimes.* In the fifth simulation study, we also employ Spec-EM (EM algorithm initialized by spectral clustering), motivated by the fact that when the number of items is moderately large, spectral clustering provides an effective initialization for the classical EM algorithm for LCMs. In particular, we consider the following regimes: (i) N=J (Figure 6), (ii) N=J/2 (Figure 7), (iii) fix N=100 and vary *J* (Figure 8), (iv) fix J=100 and vary *N* (Figure 9), (v) fix J=30 and vary *N* (Figure 10), and (vi) N=J with $\left.l,b,r,a,ces_{i}^{⋆},r,b,r,a\right.ce_{i=1}^{N}$ being independently generated from 3 with probability $\left(p_{1},p_{2},p_{3})=(1/6,1/3,1/2\right)$ (Figure 11). In all these settings, we set $\left(\beta_{1},\beta_{2})=(1,8\right)$ . In addition, we consider (vii) N=J under the fixed design of Θ (Figure 12), where the fixed item parameter matrix Θ is constructed by vertically concatenating copies of a 5×3 block matrix $\Theta_{0}$ as follows:

$$\begin{array}{c} \Theta =\left[\begin{array}{ccccc} \backslash boldsymbol & \Theta_{0} & \vdots & \backslash boldsymbol & \Theta_{0} \end{array}\right]\backslash rbrace\sim b\sim \text{copies},\;\Theta_{0}:=\left[\begin{array}{ccccccccccccccc} 0.3 & 0.7 & 0.7 & 0.3 & 0.7 & 0.3 & 0.7 & 0.3 & 0.7 & 0.7 & 0.3 & 0.3 & 0.7 & 0.7 & 0.3 \end{array}\right]. \end{array}$$

Here, the block matrix $\Theta_{0}$ is vertically stacked *b* times to yield J=5b items with K=3 latent classes. It is clear that SOLA and SOLA+ perform significantly better in most scenarios except the most traditional large-*N*, small-*J* regime, where Spec-EM performs better. This underpins both the validity of SOLA and spectral clustering.

*Simulation 6: Estimation of number of latent classes.* Our theoretical guarantee for the selection criterion of *K* relies on random matrix theory under the asymptotic regime N,J→∞ . This means relatively large *N* and *J* are needed for the theoretical guarantee to hold, which is consistent with our focus on high-dimensional data. The constant “2” in the threshold reflects a worst-case bound and can indeed be conservative, especially when entries of Θ are mostly concentrated away from 1/2 . To illustrate its effectiveness in practice, we have performed simulation studies with N=2J and K=3 , under several generative mechanisms for Θ (i.i.d. Beta (1,1) , Beta (1,4) , and Beta (1,5) , which reflect different sparsity levels, and also the fixed design as described in Simulation 5). As shown in Table 3, the criterion successfully recovers *K* in high-dimensional regimes. Our real-data analysis in Section 5.2 also demonstrates that the method works well when the data are sufficiently large. For smaller *N* and *J*, we recommend that practitioners use classical information criteria, such as BIC, as a more reliable and interpretable tool for selecting *K*.

**Table 3:** Proportion of successfully selecting $\hat{K}$ to be the true *K* based on 200 simulation replicates, under different generative mechanisms of the item parameters $\Theta ={\left(\theta_{j,k}\right)}_{J\times K}$ Table 3 long description.

*J*	1,000	1,200	1,400	1,600	1,800	2,000
Fixed design of item parameters	100\%	100\%	100\%	100\%	100\%	100\%
$\theta_{j,k}\backslash stackreli.i.d.∼$ Beta (1,1)	100\%	100\%	100\%	100\%	100\%	100\%
$\theta_{j,k}\backslash stackreli.i.d.∼$ Beta (1,4)	0\%	0\%	100\%	100\%	100\%	100\%
$\theta_{j,k}\backslash stackreli.i.d.∼$ Beta (1,5)	0\%	0\%	0\%	0\%	70\%	100\%

### 5.2 Real data example

We further evaluate the performance of our methods on the United States 112th Senate Roll Call Votes data, which is publicly available at https://legacy.voteview.com/senate112.htm. The dataset contains voting records for J=486 roll calls by 102 U.S. senators. Following the preprocessing procedure in Lyu et al. (2025), we convert the response matrix to binary and remove senators who are neither Democrats nor Republicans, as well as those with more than 10\% missing votes. This results in a final sample of N=94 senators. For the remaining missing entries, we impute values by randomly assigning 0 or 1 with probability equal to the positive response rate observed from each senator’s non-missing votes.

We compare the performance of the methods considered in our simulation studies. Table 4 summarizes the mis-clustering error and computation time (in seconds) for each method. Interestingly, the SVD approach yields an error comparable to those obtained with SOLA and SOLA+, suggesting that the refinement step may be unnecessary for this dataset. This observation can be partially explained by our remark following Theorem 2: when the number of items *J* is substantially larger than the number of individuals *N*, spectral clustering alone can perform very well. In contrast, the traditional EM method exhibits significantly higher mis-clustering error, and EM and Tensor-CEM suffer from high computational cost in this large-*J* regime.

**Table 4:** Mis-clustering error and running time (seconds) of different methods on Senate Roll Call Votes data Table 4 long description.

	SVD	SOLA	SOLA+	EM	Tensor-CEM
Error	0.0212	0.0212	0.0212	0.117	0.0212
Time	0.00179	0.0344	0.0503	3.125	5.154

We also validate the performance of our method for estimating *K* on this real dataset. In particular, the threshold defined in (17) is $2.01\times (\sqrt{486}+\sqrt{94})\approx 63.8$ . The first three largest singular values of R are {148.1,64.4,16.6} , which leads to an estimated number of clusters $\hat{K}=2$ . Notably, this estimated value exactly matches the known number of clusters in the dataset (i.e., K=2 ).

## 6 Discussion

In this work, we have proposed SOLA, a simple yet powerful two-stage algorithm, under binary-response LCMs. Our method efficiently exploits the low-rank structure of the response matrix and further leverages the likelihood information. We have proved that SOLA attains the optimal mis-clustering rate and achieves the fundamental statistical limit. We have also empirically demonstrated its superior accuracy, stability, and speed compared to other methods.

Several interesting directions remain for future research. Many applications involve multivariate polytomous responses with more than two categories for each item. Extending SOLA to handle polytomous-response LCMs is an important future direction. It remains intriguing to investigate how to modify the algorithm accordingly and obtain similar theoretical guarantees. In addition, beyond estimation, practitioners often also want to conduct statistical inference on item parameters Θ . It is worth exploring that whether we can build on SOLA to develop an estimator of item parameters Θ together with confidence intervals.

## A Appendix of the proofs

We present a general version of Proposition 3. To this end, we introduce some additional notation. For ∀j∈J and $k_{1}\neq k_{2}\in K$ , define $\Delta_{j,k_{1},k_{2}}^{2}:={\left(\theta_{j,k_{1}}-\theta_{j,k_{2}}\right)}^{2}/\left[8(\sigma_{\theta_{j,k_{1}}}^{2}\vee \sigma_{\theta_{j,k_{2}}}^{2})\right]$ . We have the following result, whose proof can be found in Section B.5. Proposition A.1. For any $k_{1}\neq k_{2}\in K$ , define

$$\begin{array}{c} \tau_{k_{1},k_{2}}:=\frac{\sum_{j\in \Omega_{0}\left(k_{1},k_{2}\right)}\Delta_{j,k_{1},k_{2}}^{2}\cdot \tau \left(\theta_{j,k_{1}}\right)}{\sum_{j\in \Omega_{0}\left(k_{1},k_{2}\right)}\Delta_{j,k_{1},k_{2}}^{2}}\left(1+\eta \right),\;\tau (x)=\frac{1-2x}{2x(1-x)\log\left(\left(1-x\right)/x\right)}, \end{array}$$

for some η=o(1) . Assume that (a) $\max_{k_{1}\neq k_{2}\in K}\left|\Omega_{0}^{c}(k_{1},k_{2})\right|=O(1),$ where $\Omega_{0}(k_{1},k_{2}):=\left.l,b,r,a,cej\in J:\left|\theta_{j,k_{1}}-\theta_{j,k_{2}}\right|=o(1),r,b,r,a\right.ce$ , then we have

\exp−I⋆2≲\exp−Δ28^¯σ21+τ_,

where $\underline{\tau }:=\min_{k_{1}\neq k_{2}\in K}\tau_{k_{1},k_{2}}$ . Furthermore, if (b) $\min_{k\in K}\left|\left.l,b,r,a,cej\in J:\left|\theta_{j,k}-1/2\right|>c_{0},r,b,r,a\right.ce\right|≳J$ for some universal constant $c_{0}>0$ , then we have $\underline{\tau }>c_{\tau }$ for some universal constant $c_{\tau }>0$ .

## B Proofs of main results

### B.1 Proof of Theorem 1

The proof can be adapted from Theorem S.4 in Lyu et al. (2025) by fixing the individual degree parameters therein to be all 1 ’s. We only sketch essential modifications here. Let

$$\begin{array}{c} P_{\Theta }:=\left.l,b,r,a,ce\tilde{\Theta }:\tilde{\theta_{j,k}}\in c_{\theta },C_{\theta },j\in J,k\in K,\max_{j\in J}\max_{k\neq k^{\prime }}\left|\tilde{\theta_{j,k}}-\tilde{\theta_{j,k^{\prime }}}\right|=o(1),r,b,r,a\right.ce. \end{array}$$

Following the construction of $V^{*}$ and the proof of Theorem S.4 in Lyu et al. (2025), it suffices to consider the following space:

$$\begin{array}{c} \bar{\_}PK\left(s,\Theta \right):=\left.l,b,r,a,ce\bar{R}:\bar{\_}Ri,j=\theta_{j,s_{i}},s\in V^{*},\Theta \in P_{\Theta },r,b,r,a\right.ce. \end{array}$$

Then we can follow the proof of Theorem S.4 in Lyu et al. (2025). Notice that in the proof of Lemma S.2, the argument therein still holds by allowing $p_{1,j}≍p_{2,j}≍1$ and $\left|p_{1,j}-p_{2,j}\right|=o\left(p_{1,j}\right)$ . We can then get the desired result by directly applying the modified Lemma S.2 without further lower bounding it.

### B.2 Proof of Theorem 2

*Initialization:* We start with investigating the performance of $\hat{\theta_{j,k}^{\left(m\right)}}$ ’s. To this end, for any ρ=o(1) , we define the event

(B.1)
$$\begin{array}{c} E_{\rho ,m}:=\left.l,b,r,a,ce\min_{\pi \in G_{K}}\max_{j\in J,k\in K}\left|\hat{\theta_{j,k}^{\left(m\right)}}-\theta_{j,\pi \left(k\right)}\right|\leq \rho I^{⋆}/J,r,b,r,a\right.ce. \end{array}$$

The following lemma indicates that (B.1) holds with high probability, whose proof is deferred to Section C.1. Lemma B.1. There exists some absolute constant $c_{0}\in (0,1)$ such that P(Eρ,m)≥1−\exp(−(1−c0)Δ2/(8^¯σ2)) for m∈2 with ρ=o(1/K) .

*One-step refinement:* In the following, we will derive the clustering error for $i\in S_{1}$ . Fix any $k\in K\backslash \left.l,b,r,a,ces_{i}^{⋆},r,b,r,a\right.ce$ . Without loss of generality, we assume that π=Id (identity map with π(k)=k for all *k*) in (B.1) as otherwise we can replace $\theta_{j,k}$ with $\theta_{j,\pi (k)}$ and $\theta_{j,s_{i}^{*}}$ with $\theta_{j,\pi (s_{i}^{*})}$ in the following derivation. By definition, we have

(B.2)
$$\begin{array}{ccccc} I\left(\hat{s_{i}}=k\right) & \leq I\left(\sum_{j\in J}R_{i,j}\log\frac{\hat{\theta_{j,k}^{\left(2\right)}}\left(1-\hat{\theta_{j,s_{i}^{⋆}}^{\left(2\right)}}\right)}{\hat{\theta_{j,s_{i}^{⋆}}^{\left(2\right)}}\left(1-\hat{\theta_{j,k}}\right)}\geq \sum_{j\in J}\log\frac{1-\hat{\theta_{j,s_{i}^{⋆}}^{\left(2\right)}}}{1-\hat{\theta_{j,k}^{\left(2\right)}}}\right) & \leq I\left(\sum_{j\in J}R_{i,j}\log\frac{\theta_{j,k}\left(1-\theta_{j,s_{i}^{⋆}}\right)}{\theta_{j,s_{i}^{⋆}}\left(1-\theta_{j,k}\right)}\geq \sum_{j\in J}\log\frac{1-\theta_{j,s_{i}^{⋆}}}{1-\theta_{j,k}}-\delta I^{⋆}\right) & \;+I(\sum_{j\in J}R_{i,j}\left(\log\frac{\hat{\theta_{j,k}^{\left(2\right)}}\left(1-\hat{\theta_{j,s_{i}^{⋆}}^{\left(2\right)}}\right)}{\hat{\theta_{j,s_{i}^{⋆}}^{\left(2\right)}}\left(1-\hat{\theta_{j,k}^{\left(2\right)}}\right)}-\log\frac{\theta_{j,k}\left(1-\theta_{j,s_{i}^{⋆}}\right)}{\theta_{j,s_{i}^{⋆}}\left(1-\theta_{j,k}\right)}\right) & \geq \sum_{j\in J}\left(\log\frac{1-\hat{\theta_{j,s_{i}^{⋆}}^{\left(2\right)}}}{1-\hat{\theta_{j,k}^{\left(2\right)}}}-\log\frac{1-\theta_{j,s_{i}^{⋆}}}{1-\theta_{j,k}}\right)+\delta I^{⋆}), \end{array}$$

where δ=o(1) is some sequence to be determined later. We start with bounding the first term in (B.2). By the Chernoff bound, we obtain that

(B.3)
$$\begin{array}{cccc} P & \left(\sum_{j\in J}R_{i,j}\log\frac{\theta_{j,k}\left(1-\theta_{j,s_{i}^{⋆}}\right)}{\theta_{j,s_{i}^{⋆}}\left(1-\theta_{j,k}\right)}\geq \sum_{j\in J}\log\frac{1-\theta_{j,s_{i}^{⋆}}}{1-\theta_{j,k}}-\delta I^{⋆}\right) & \leq \backslash exp\left(-\sum_{j\in J}\log\sqrt{\frac{1-\theta_{j,s_{i}^{⋆}}}{1-\theta_{j,k}}}+\frac{\delta I^{⋆}}{2}\right)\prod_{j=1}^{J}\left(\sqrt{\theta_{j,s_{i}^{⋆}}\theta_{j,k}}\sqrt{\frac{1-\theta_{j,s_{i}^{⋆}}}{1-\theta_{j,k}}}+1-\theta_{j,s_{i}^{⋆}}\right) & \leq \backslash exp\left(\frac{\delta I^{⋆}}{2}\right)\backslash exp\left(-\frac{I^{⋆}}{2}\right)=\backslash exp\left(-\frac{I^{⋆}}{2}\left(1-\delta \right)\right). \end{array}$$

Using this, we can bound the second term in (B.2) as

(B.4)
$$\begin{array}{cccc} & I\left(\sum_{j\in J}R_{i,j}\left(\log\frac{\hat{\theta_{j,k}^{\left(2\right)}}\left(1-\hat{\theta_{j,s_{i}^{⋆}}^{\left(2\right)}}\right)}{\hat{\theta_{j,s_{i}^{⋆}}^{\left(2\right)}}\left(1-\hat{\theta_{j,k}^{\left(2\right)}}\right)}-\log\frac{\theta_{j,k}\left(1-\theta_{j,s_{i}^{⋆}}\right)}{\theta_{j,s_{i}^{⋆}}\left(1-\theta_{j,k}\right)}\right)\geq \sum_{j\in J}\left(\log\frac{1-\hat{\theta_{j,s_{i}^{⋆}}^{\left(2\right)}}}{1-\hat{\theta_{j,k}^{\left(2\right)}}}-\log\frac{1-\theta_{j,s_{i}^{⋆}}}{1-\theta_{j,k}}\right)+\delta I^{⋆}\right) & \leq I\left(\sum_{j\in J}R_{i,j}\log\frac{\hat{\theta_{j,k}^{\left(2\right)}}}{\theta_{j,k}}>\frac{\delta }{4}I^{⋆}\right)+I\left(\sum_{j\in J}R_{i,j}\log\frac{\theta_{j,s_{i}^{⋆}}}{\hat{\theta_{j,s_{i}^{⋆}}}}>\frac{\delta }{4}I^{⋆}\right) & \;+I\left(\sum_{j\in J}\left(R_{i,j}-1\right)\log\frac{1-\hat{\theta_{j,s_{i}^{⋆}}^{\left(2\right)}}}{1-\theta_{j,s_{i}^{⋆}}}>\frac{\delta }{4}I^{⋆}\right)+I\left(\sum_{j\in J}\left(R_{i,j}-1\right)\log\frac{1-\theta_{j,k}}{1-\hat{\theta_{j,k}^{\left(2\right)}}}>\frac{\delta }{4}I^{⋆}\right). \end{array}$$

For the first term in (B.4), we proceed by noticing that

(B.5)
$$\begin{array}{ccc} & I\left(\sum_{j\in J}R_{i,j}\log\frac{\hat{\theta_{j,k}^{\left(2\right)}}}{\theta_{j,k}}>\frac{\delta }{4}I^{⋆}\right) & \leq I\left(\sum_{j\in J}R_{i,j}\log\frac{\hat{\theta_{j,k}^{\left(2\right)}}}{\theta_{j,k}}I\left(E_{\rho ,2}\right)>\frac{\delta }{8}I^{⋆}\right)+I\left(\sum_{j\in J}R_{i,j}\log\frac{\hat{\theta_{j,k}^{\left(2\right)}}}{\theta_{j,k}}I\left(E_{\rho ,2}^{c}\right)>\frac{\delta }{8}I^{⋆}\right). \end{array}$$

Let $\delta =\rho^{\epsilon }$ for ϵ∈(0,1) and $\lambda =c\rho^{-\epsilon }$ for some constant c>0 depending only on $c_{\theta }$ and $C_{\theta }$ , then by Chernoff bound on the first term of (B.5), we obtain that

$$\begin{array}{cccc} & P\left(\sum_{j\in J}R_{i,j}\log\frac{\hat{\theta_{j,k}^{\left(2\right)}}}{\theta_{j,k}}I\left(E_{\rho ,2}\right)>\frac{\delta }{8}I^{⋆}\right)\backslash overset\left(a\right)\leq P\left(\sum_{j\in J}R_{i,j}\frac{\rho I^{⋆}}{J}>\frac{c_{\theta }\delta }{8}I^{⋆}\right) & \leq \backslash exp\left(-\lambda \frac{c_{\theta }\delta }{8}I^{⋆}\right)\prod_{j=1}^{J}E\backslash exp\left(\lambda R_{i,j}\frac{\rho I^{⋆}}{J}\right)\backslash overset\left(b\right)\leq \backslash exp\left(-\lambda \frac{c_{\theta }\delta }{8}I^{⋆}\right)\prod_{j=1}^{J}\left(1+2\lambda \theta_{i,j}\frac{\rho I^{⋆}}{J}\right) & \leq \backslash exp\left(-\lambda \frac{c_{\theta }\delta }{8}I^{⋆}\right)\backslash exp\left(\rho \cdot 2C_{\theta }I^{⋆}\right)\backslash overset\left(c\right)\leq \backslash exp\left(-\frac{I^{⋆}}{2}\right), \end{array}$$

where (a) holds due to (B.1) and log(1+x)≤x for x>−1 , (b) holds due to $e^{x}-1\leq 2x$ for 0<x<1 and the fact that $\lambda \rho I^{⋆}/J≲\rho^{1-\epsilon }\Delta^{2}/J=o(1)$ , and (c) holds due to the choice of $\lambda =c\rho^{-\varepsilon }$ . For the second term of (B.5), by the independence between $\hat{\theta_{j,k}^{\left(2\right)}}$ and $\left.l,b,r,a,ceR_{i,j},j\in J,r,b,r,a\right.ce$ , we get

$$\begin{array}{ccc} P & \left(\sum_{j\in J}R_{i,j}\log\frac{\hat{\theta_{j,k}^{\left(2\right)}}}{\theta_{j,k}}I\left(E_{\rho ,2}^{c}\right)>\frac{\delta }{8}I^{⋆}\right)\leq \backslash exp\left(-\lambda \frac{\delta }{8}I^{⋆}\right)\prod_{j=1}^{J}E\backslash exp\left(\lambda R_{i,j}\log\frac{\hat{\theta_{j,k}^{\left(2\right)}}}{\theta_{j,k}}I\left(E_{\rho ,2}^{c}\right)\right) & \leq \backslash exp\left(-\lambda \frac{\delta }{8}I^{⋆}\right)\prod_{j=1}^{J}\left[1+\theta_{j,k}\left(E\backslash exp\left(\lambda \log\frac{\hat{\theta_{j,k}^{\left(2\right)}}}{\theta_{j,k}}I\left(E_{\rho ,2}^{c}\right)\right)-1\right)\right]. \end{array}$$

To bound the above term, notice that

E\expλlogθj,k(2)^θj,kIEρ,2c−1≤\expλlog1cθ−1PEρ,2c≤\expλlog1cθ−Δ216^¯σ2.

Since we can always choose ρ→0 sufficiently slow such that

λlog1cθ≲1ρε≲Δ2^¯σ2≍I⋆,

we can conclude that

$$\begin{array}{ccc} P & \left(\sum_{j\in J}R_{i,j}\log\frac{\hat{\theta_{j,k}^{\left(2\right)}}}{\theta_{j,k}}I\left(E_{\rho ,2}^{c}\right)>\frac{\delta }{8}I^{⋆}\right)\leq \backslash exp\left(-\lambda \frac{\delta }{8}I^{⋆}\right)\prod_{j=1}^{J}\left[1+\theta_{j,k}\backslash exp\left(-cI^{⋆}\right)\right] & \leq \backslash exp\left(-\lambda \frac{\delta }{8}I^{⋆}\right){\left(1+\frac{C_{\theta }}{\backslash exp\left(cI^{⋆}\right)}\right)}^{J}\leq \backslash exp\left(-\frac{I^{⋆}}{2}\right). \end{array}$$

where the last inequality due to the fact that ${\left(1+C_{\theta }\backslash exp(-cI^{⋆})\right)}^{J}=O(1)$ provided that $\backslash exp(cI^{⋆})\geq J$ . We thereby arrive at

(B.6)
$$\begin{array}{c} P\left(\sum_{j\in J}R_{i,j}\log\frac{\hat{\theta_{j,k}^{\left(2\right)}}}{\theta_{j,k}}>\frac{\delta }{4}I^{⋆}\right)\leq 2\backslash exp\left(-\frac{I^{⋆}}{2}\right). \end{array}$$

The second term in (B.4) can be bounded in the same fashion. For the third term in (B.4), we have

$$\begin{array}{ccc} & I\left(\sum_{j\in J}\left(R_{i,j}-1\right)\log\frac{1-\hat{\theta_{j,s_{i}^{⋆}}^{\left(2\right)}}}{1-\theta_{j,s_{i}^{⋆}}}>\frac{\delta }{4}I^{⋆}\right) & \leq I\left(\sum_{j\in J}\left(R_{i,j}-1\right)\log\frac{1-\hat{\theta_{j,s_{i}^{⋆}}^{\left(2\right)}}}{1-\theta_{j,s_{i}^{⋆}}}I\left(E_{\rho ,2}\right)>\frac{\delta }{8}I^{⋆}\right)+I\left(\sum_{j\in J}\left(R_{i,j}-1\right)\log\frac{1-\hat{\theta_{j,s_{i}^{⋆}}^{\left(2\right)}}}{1-\theta_{j,s_{i}^{⋆}}}I\left(E_{\rho ,2}^{c}\right)>\frac{\delta }{8}I^{⋆}\right). \end{array}$$

Similarly, we can bound the expectation of the first term in the above equation as

$$\begin{array}{ccccc} & P\left(\sum_{j\in J}\left(R_{i,j}-1\right)\log\frac{1-\hat{\theta_{j,s_{i}^{⋆}}^{\left(2\right)}}}{1-\theta_{j,s_{i}^{⋆}}}I\left(E_{\rho ,2}\right)>\frac{\delta }{8}I^{⋆}\right) & \leq \backslash exp\left(-\lambda \frac{\left(1-C_{\theta }\right)\delta }{8}I^{⋆}\right)\prod_{j=1}^{J}E\backslash exp\left(\lambda \left(R_{i,j}-1\right)\frac{\rho I^{*}}{J}\right) & \backslash overset\left(a\right)\leq \backslash exp\left(-\lambda \frac{\left(1-C_{\theta }\right)\delta }{8}I^{⋆}\right)\prod_{j=1}^{J}\left(1+\frac{C_{\theta }\rho I^{*}}{2J}\right) & \leq \backslash exp\left(-\lambda \frac{\left(1-C_{\theta }\right)\delta }{8}I^{⋆}\right)\backslash exp\left(\rho \cdot \frac{C_{\theta }I^{*}}{2}\right)\leq \backslash exp\left(-\frac{I^{⋆}}{2}\right), \end{array}$$

where (a) holds due to $e^{-x}\leq 1-x/2$ for x∈0,1 . Similarly, we have that

$$\begin{array}{cccccc} & P\left(\sum_{j\in J}\left(R_{i,j}-1\right)\log\frac{1-\hat{\theta_{j,s_{i}^{⋆}}^{\left(2\right)}}}{1-\theta_{j,s_{i}^{⋆}}}I\left(E_{\rho ,2}^{c}\right)>\frac{\delta }{8}I^{⋆}\right) & \leq \backslash exp\left(-\lambda \frac{\delta }{8}I^{⋆}\right)\prod_{j=1}^{J}\left[\theta_{j,s_{i}^{*}}+\left(1-\theta_{j,s_{i}^{*}}\right)E\backslash exp\left(\lambda \log\frac{1-\hat{\theta_{j,s_{i}^{*}}^{\left(2\right)}}}{1-\theta_{j,s_{i}^{*}}}I\left(E_{\rho ,2}^{c}\right)\right)\right] & \leq \backslash exp\left(-\lambda \frac{\delta }{8}I^{⋆}\right)\prod_{j=1}^{J}\left[\theta_{j,s_{i}^{*}}+\left(1-\theta_{j,s_{i}^{*}}\right)\backslash exp\left(\lambda \log\frac{1}{1-C_{\theta }}\right)P\left(E_{\rho ,2}^{c}\right)\right] & \leq \backslash exp\left(-\lambda \frac{\delta }{8}I^{⋆}\right)\prod_{j=1}^{J}\left[\theta_{j,s_{i}^{*}}+\left(1-\theta_{j,s_{i}^{*}}\right)\backslash exp\left(-cI^{*}\right)\right] & \leq \backslash exp\left(-\lambda \frac{\delta }{8}I^{⋆}\right)C_{\theta }^{J}{\left(1+\frac{1}{c_{\theta }\backslash exp\left(cI^{*}\right)}\right)}^{J}\leq \backslash exp\left(-\frac{I^{⋆}}{2}\right), \end{array}$$

provided that $\backslash exp(cI^{⋆})\geq J$ . We thus get that

(B.7)
$$\begin{array}{c} P\left(\sum_{j\in J}\left(R_{i,j}-1\right)\log\frac{1-\hat{\theta_{j,s_{i}^{⋆}}^{\left(2\right)}}}{1-\theta_{j,s_{i}^{⋆}}}>\frac{\delta }{4}I^{⋆}\right)\leq 2\backslash exp\left(-\frac{I^{⋆}}{2}\right). \end{array}$$

The last term in (B.4) can be bounded in the same fashion. By (B.2), (B.3), (B.4), (B.6), and (B.7), we thereby arrive at

$$\begin{array}{c} P\left(\hat{s_{i}}=k\right)\leq \backslash exp\left(-\frac{I^{⋆}}{2}\left(1-\rho^{\epsilon }\right)\right)+8\backslash exp\left(-\frac{I^{⋆}}{2}\right)\leq \backslash exp\left(-\frac{I^{⋆}}{2}\left(1-o(1)\right)\right). \end{array}$$

We thus conclude that

$$\begin{array}{c} \frac{1}{\left|S_{1}\right|}E\sum_{i\in S_{1}}I\left(\hat{s_{i}}\neq s_{i}^{⋆}\right)\leq \backslash exp\left(-\frac{I^{⋆}}{2}\left(1-o(1)\right)\right). \end{array}$$

The proof for the case $i\in S_{2}$ can be conducted in the same fashion. We can conclude that

$$\begin{array}{c} \frac{1}{\left|S_{2}\right|}E\sum_{i\in S_{2}}I\left(\hat{s_{i}}\neq \pi_{0}\left(s_{i}^{⋆}\right)\right)\leq \backslash exp\left(-\frac{I^{⋆}}{2}\left(1-o(1)\right)\right) \end{array}$$

for some $\pi_{0}\in G_{K}$ .

*Label alignment:* We then proceed with the proof for label alignment. Recall that without loss of generality, we have assumed that $\ell (\tilde{s^{\left(1\right)}},s_{S_{1}}^{⋆})=\ell_{0}(\tilde{s^{\left(1\right)}},s_{S_{1}}^{⋆})$ and $\ell (\tilde{s^{\left(2\right)}},s_{S_{2}}^{⋆})=\ell_{0}(\tilde{s^{\left(2\right)}},\pi_{0}(s_{S_{2}}^{⋆}))$ for some $\pi_{0}\in G_{K}$ . Notice that

$$\begin{array}{c} \ell_{0}\left(\hat{s_{S_{2}}},s_{S_{2}}^{⋆}\right)=\ell_{0}\left(\hat{\pi }(\hat{s^{\left(2\right)}}),s_{S_{2}}^{⋆}\right)=\ell_{0}\left(\hat{s^{\left(2\right)}},\hat{\pi^{-1}}(s_{S_{2}}^{⋆})\right). \end{array}$$

It suffices to show that $\hat{\pi }=\pi_{0}^{-1}$ , then we have

$$\begin{array}{c} E\ell_{0}\left(\hat{s^{\left(2\right)}},\hat{\pi^{-1}}(s_{S_{2}}^{⋆})\right)=E\ell_{0}\left(\hat{s^{\left(2\right)}},\pi_{0}(s_{S_{2}}^{⋆})\right)\leq \backslash exp\left(-\frac{I^{⋆}}{2}\left(1-o(1)\right)\right), \end{array}$$

as desired. By definition, we have

$$\begin{array}{ccc} {\left\|\hat{\Theta^{\left(1\right)}}-\hat{\Theta^{\left(2\right)}}G_{\pi_{0}^{-1}}\right\|}_{F}^{2} & =\sum_{j\in J}\sum_{k\in K}{\left|\hat{\theta_{j,k}^{\left(1\right)}}-\hat{\theta_{j,\pi_{0}^{-1}(k)}^{\left(2\right)}}\right|}^{2} & \leq \sum_{j\in J}\sum_{k\in K}{\left|\hat{\theta_{j,k}^{\left(1\right)}}-\theta_{j,k}\right|}^{2}+\sum_{j\in J}\sum_{k\in K}{\left|\hat{\theta_{j,\pi_{0}^{-1}(k)}^{\left(2\right)}}-\theta_{j,k}\right|}^{2}\leq 2\rho KI^{⋆}. \end{array}$$

On the other hand, for any $\pi \in G_{K}$ and $\pi \neq \pi_{0}^{-1}$ , we have

$$\begin{array}{ccc} {\left\|\hat{\Theta^{\left(1\right)}}-\hat{\Theta^{\left(2\right)}}G_{\pi }\right\|}_{F}^{2} & =\sum_{j\in J}\sum_{k\in K}{\left|\hat{\theta_{j,k}^{\left(1\right)}}-\hat{\theta_{j,\pi (k)}^{\left(2\right)}}\right|}^{2} & \geq \sum_{j\in J}\sum_{k\in K}{\left|\hat{\theta_{j,\pi (k)}^{\left(2\right)}}-\theta_{j,k}\right|}^{2}-\rho KI^{⋆}. \end{array}$$

It suffices to show that $\sum_{j\in J}\sum_{k\in K}{\left|\hat{\theta_{j,\pi (k)}^{\left(2\right)}}-\theta_{j,k}\right|}^{2}>4\rho KI^{⋆}$ . Observe that

$$\begin{array}{ccc} \left|\hat{\theta_{j,\pi (k)}^{\left(2\right)}}-\theta_{j,k}\right| & =\left|\hat{\theta_{j,\pi (k)}^{\left(2\right)}}-\theta_{j,\pi \circ \pi_{0}(k)}+\theta_{j,\pi \circ \pi_{0}(k)}-\theta_{j,k}\right| & \geq \left|\theta_{j,\pi \circ \pi_{0}(k)}-\theta_{j,k}\right|-\left|\hat{\theta_{j,\pi (k)}^{\left(2\right)}}-\theta_{j,\pi \circ \pi_{0}(k)}\right|. \end{array}$$

Notice that $\left|\hat{\theta_{j,\pi (k)}^{\left(2\right)}}-\theta_{j,\pi \circ \pi_{0}(k)}\right|=\left|\hat{\theta_{j,k}^{\left(2\right)}}-\theta_{j,\pi_{0}(k)}\right|\leq \rho I^{⋆}/J$ , we have

$$\begin{array}{ccccc} & \sum_{j=1}^{J}\sum_{k=1}^{K}{\left(\hat{\theta_{j,\pi (k)}^{\left(2\right)}}-\theta_{j,k}\right)}^{2} & \geq \sum_{j=1}^{J}\sum_{k=1}^{K}{\left(\theta_{j,\pi \circ \pi_{0}(k)}-\theta_{j,k}\right)}^{2}-2\sum_{j=1}^{J}\sum_{k=1}^{K}\left|\theta_{j,\pi \circ \pi_{0}(k)}-\theta_{j,k}\right|\left|\hat{\theta_{j,\pi (k)}^{\left(2\right)}}-\theta_{j,\pi \circ \pi_{0}(k)}\right| & \geq \sum_{j=1}^{J}\sum_{k=1}^{K}{\left(\theta_{j,\pi \circ \pi_{0}(k)}-\theta_{j,k}\right)}^{2}-\frac{2\rho I^{⋆}}{J}\sum_{j=1}^{J}\sum_{k=1}^{K}\left|\theta_{j,\pi \circ \pi_{0}(k)}-\theta_{j,k}\right| & \geq \left(\sum_{k=1}^{K}I\left(\pi \circ \pi_{0}(k)\neq k\right)\right)\left(\Delta^{2}-4C_{\theta }\rho I^{⋆}\right)\geq \Delta^{2}, \end{array}$$

where the last inequality holds due to the fact that $\pi \neq \pi_{0}^{-1}$ , $\Delta^{2}≍I^{⋆}\to \infty ,$ and ρ=o(1) . The proof is thus completed by using ρ=o(1/K) .

### B.3 Proof of Proposition 2

Notice that $\lambda_{K}\geq \lambda_{K}\left(Z\right)\lambda_{K}\left(\Theta \right)$ . To get a lower bound for $\lambda_{K}$ , it suffices to obtain lower bounds for $\lambda_{K}(Z)$ and $\lambda_{K}(\Theta )$ . First note that

$$\begin{array}{c} Z^{⊤}Z=\text{diag}\left(\left|\left.l,b,r,a,cei:s_{i}^{⋆}=1,r,b,r,a\right.ce\right|,\cdots ,\left|\left.l,b,r,a,cei:s_{i}^{⋆}=K,r,b,r,a\right.ce\right|\right). \end{array}$$

We thus obtain $\lambda_{K}(Z)\geq \sqrt{\alpha N/K}$ . On the other hand, by Proposition 2 in Lyu et al. (2025), we obtain that for any δ∈(0,1) ,

$$\begin{array}{c} P\left(\lambda_{K}\left(\Theta^{⊤}\Theta \right)\geq \left(1-\delta \right)BJK\right)\leq K\left(e^{-\delta^{2}B^{2}J/16}+e^{-3\delta BJ/8}\right). \end{array}$$

Denote the above event as $E_{\theta }$ , under which we arrive at $\lambda_{K}(\Theta )\geq (1-\delta /2)\sqrt{BJK}$ .

To obtain a lower bound for Δ , it suffices to use Lemma S.1 in Lyu et al. (2025) to get $\Delta \geq \sqrt{2}\lambda_{K}(\Theta )$ . We get the desired result by setting δ=1/2 .

### B.4 Proof of Corollary 1

The proof follows the same argument as that of Theorem 2. We only sketch the modifications here. Without loss of generality, we only consider sample data points in $S_{1}$ . First note that by Proposition 3.1 in Zhang and Zhou (2024), we have $\ell (\tilde{s^{\left(2\right)}},s_{S_{2}}^{⋆})=o(1)$ with probability at least $1-O(e^{-N\vee J})$ . Hence, we get that

$$\begin{array}{c} \log\left(\frac{\hat{p_{k}}}{\hat{\pi_{s_{i}^{⋆}}}}\right)=\log\left(\frac{\hat{p_{k}}}{p_{k}}\right)+\log\left(\frac{\pi_{s_{i}^{⋆}}}{\hat{\pi_{s_{i}^{⋆}}}}\right)+\log\left(\frac{p_{k}}{\pi_{s_{i}^{⋆}}}\right)≲\log\left(\frac{1}{\alpha }\right)=o\left(I\right) \end{array}$$

with probability at least $1-O(e^{-N\vee J})$ , where we’ve used Assumption 2. This indicates that the impact of this additional term is ignorable, that is,

$$\begin{array}{c} P\left(\log\left(\frac{\hat{p_{k}}}{\hat{\pi_{s_{i}^{⋆}}}}\right)>c\delta I^{⋆}\right)\leq O\left(e^{-N\vee J}\right) \end{array}$$

for some universal constant c>0 . The remaining proof follows from that of Theorem 2 line by line.

### B.5 Proof of Proposition 4

Specifically, we fix $j\in \Omega_{0}(k_{1},k_{2})$ and $k_{1}\neq k_{2}\in K$ in the following analysis. Note that

$$\begin{array}{cccc} & -2\log\left(\sqrt{\theta_{j,k_{1}}\theta_{j,k_{2}}}+\sqrt{\left(1-\theta_{j,k_{1}}\right)\left(1-\theta_{j,k_{2}}\right)}\right) & =-\log\left(1-{\left(\sqrt{\theta_{j,k_{1}}}-\sqrt{\theta_{j,k_{2}}}\right)}^{2}-2\sqrt{\theta_{j,k_{1}}\theta_{j,k_{2}}}\left(1-\sqrt{\theta_{j,k_{1}}\theta_{j,k_{2}}}-\sqrt{\left(1-\theta_{j,k_{1}}\right)\left(1-\theta_{j,k_{2}}\right)}\right)\right) & \geq {\left(\sqrt{\theta_{j,k_{1}}}-\sqrt{\theta_{j,k_{2}}}\right)}^{2}+2\sqrt{\theta_{j,k_{1}}\theta_{j,k_{2}}}\left(1-\sqrt{\theta_{j,k_{1}}\theta_{j,k_{2}}}-\sqrt{\left(1-\theta_{j,k_{1}}\right)\left(1-\theta_{j,k_{2}}\right)}\right). \end{array}$$

When $\theta_{j,k_{1}}+\theta_{j,k_{2}}\leq 1$ , we have

$$\begin{array}{cccc} 1-\sqrt{\theta_{j,k_{1}}\theta_{j,k_{2}}}-\sqrt{\left(1-\theta_{j,k_{1}}\right)\left(1-\theta_{j,k_{2}}\right)} & =\frac{{\left(1-\sqrt{\theta_{j,k_{1}}\theta_{j,k_{2}}}\right)}^{2}-\left(1-\theta_{j,k_{1}}\right)\left(1-\theta_{j,k_{2}}\right)}{1-\sqrt{\theta_{j,k_{1}}\theta_{j,k_{2}}}+\sqrt{\left(1-\theta_{j,k_{1}}\right)\left(1-\theta_{j,k_{2}}\right)}} & =\frac{{\left(\sqrt{\theta_{j,k_{1}}}-\sqrt{\theta_{j,k_{2}}}\right)}^{2}}{1-\sqrt{\theta_{j,k_{1}}\theta_{j,k_{2}}}+\sqrt{\theta_{j,k_{1}}\theta_{j,k_{2}}+1-\theta_{j,k_{1}}-\theta_{j,k_{2}}}} & =C_{\theta_{j,k_{1}},\theta_{j,k_{2}}}{\left(\sqrt{\theta_{j,k_{1}}}-\sqrt{\theta_{j,k_{2}}}\right)}^{2.} \end{array}$$

When $\theta_{j,k_{2}}+\theta_{j,k_{1}}>1$ , we have that

$$\begin{array}{cccc} 1-\sqrt{\theta_{j,k_{1}}\theta_{j,k_{2}}}-\sqrt{\left(1-\theta_{j,k_{1}}\right)\left(1-\theta_{j,k_{2}}\right)} & =\frac{{\left(1-\sqrt{\left(1-\theta_{j,k_{1}}\right)\left(1-\theta_{j,k_{2}}\right)}\right)}^{2}-\theta_{j,k_{1}}\theta_{j,k_{2}}}{1-\sqrt{\left(1-\theta_{j,k_{1}}\right)\left(1-\theta_{j,k_{2}}\right)}+\sqrt{\theta_{j,k_{1}}\theta_{j,k_{2}}}} & =\frac{{\left(\sqrt{1-\theta_{j,k_{1}}}-\sqrt{1-\theta_{j,k_{2}}}\right)}^{2}}{1+\sqrt{\theta_{j,k_{1}}\theta_{j,k_{2}}}-\sqrt{\theta_{j,k_{1}}\theta_{j,k_{2}}-\left(\theta_{j,k_{1}}+\theta_{j,k_{2}}-1\right)}} & =C_{\theta_{j,k_{1}},\theta_{j,k_{2}}}{\left(\sqrt{1-\theta_{j,k_{1}}}-\sqrt{1-\theta_{j,k_{2}}}\right)}^{2.} \end{array}$$

Here, for any $\theta_{1},\theta_{2}\in 0,1,$ we define

$$\begin{array}{c} C_{\theta_{1},\theta_{2}}:=\backslash cases{\left(1-\sqrt{\theta_{1}\theta_{2}}+\sqrt{\theta_{1}\theta_{2}+1-\theta_{1}-\theta_{2}}\right)}^{-1},\theta_{1}+\theta_{2}\leq 1{\left(1+\sqrt{\theta_{1}\theta_{2}}-\sqrt{\theta_{1}\theta_{2}-\left(\theta_{1}+\theta_{2}-1\right)}\right)}^{-1},\theta_{1}+\theta_{2}>1. \end{array}$$

In addition, let $\bar{\_}C\theta_{1},\theta_{2}:=2C_{\theta_{1},\theta_{2}}\sqrt{\theta_{1}\theta_{2}}$ . Hence, we conclude that

$$\begin{array}{c} -2\log\left(\sqrt{\theta_{j,k_{1}}\theta_{j,k_{2}}}+\sqrt{\left(1-\theta_{j,k_{1}}\right)\left(1-\theta_{j,k_{2}}\right)}\right)\geq \left(1+\bar{\_}C\theta_{j,k_{1}},\theta_{j,k_{2}}\right)\backslash textsfD_{2}\left(\theta_{j,k_{1}},\theta_{j,k_{2}}\right), \end{array}$$

where for any $\theta_{1},\theta_{2}\in 0,1$ , we define

$$\begin{array}{c} \backslash textsfD_{2}\left(\theta_{1},\theta_{2}\right):=\backslash cases{\left(\sqrt{\theta_{1}}-\sqrt{\theta_{2}}\right)}^{2},\theta_{1}+\theta_{2}\leq 1{\left(\sqrt{1-\theta_{1}}-\sqrt{1-\theta_{2}}\right)}^{2},\theta_{1}+\theta_{2}>1. \end{array}$$

By the definition of $\Omega_{0}(k_{1},k_{2})$ , we have either (i) $\theta_{j,k_{1}}\vee \theta_{j,k_{2}}<1/2$ and $\theta_{j,k_{1}}+\theta_{j,k_{2}}\leq 1$ or (ii) $\theta_{j,k_{1}}\wedge \theta_{j,k_{2}}>1/2$ and $\theta_{j,k_{1}}+\theta_{j,k_{2}}>1$ .

We start with case (i). Without loss of generality, we assume $\frac{1}{2}>\theta_{j,k_{1}}>\theta_{j,k_{2}}$ and denote $\theta :=\theta_{2}$ , $\delta :=\theta_{1}-\theta_{2}=o(1)$ . By definition, we have

$$\begin{array}{cccc} -\log\left(\sqrt{\theta_{j,k_{1}}\theta_{j,k_{2}}}+\sqrt{\left(1-\theta_{j,k_{1}}\right)\left(1-\theta_{j,k_{2}}\right)}\right) & =\frac{\left(1+\bar{\_}C\theta +\delta ,\theta \right)}{2}{\left(\sqrt{\theta +\delta }-\sqrt{\theta }\right)}^{2}\left(1+o(1)\right) & =\delta^{2}\frac{\left(1+\bar{\_}C\theta +\delta ,\theta \right)}{8\theta }\left(1+o(1)\right) & =\frac{\delta^{2}}{8\theta \left(1-\theta \right)}\left(1+o(1)\right). \end{array}$$

On the other hand, we have

$$\begin{array}{c} \frac{{\left(\theta_{j,k_{1}}-\theta_{j,k_{2}}\right)}^{2}}{8\left(\sigma_{\theta_{j,k_{1}}}^{2}\vee \sigma_{\theta_{j,k_{1}}}^{2}\right)}=\delta^{2}\frac{\log\left(\left(1-\theta \right)/\theta \right)}{4\left(1-2\theta \right)}. \end{array}$$

This implies that

$$\begin{array}{c} \frac{-\log\left(\sqrt{\theta_{j,k_{1}}\theta_{j,k_{2}}}+\sqrt{\left(1-\theta_{j,k_{1}}\right)\left(1-\theta_{j,k_{2}}\right)}\right)}{{\left(\theta_{j,k_{1}}-\theta_{j,k_{2}}\right)}^{2}/\left(8\left(\sigma_{\theta_{j,k_{1}}}^{2}\vee \sigma_{\theta_{j,k_{1}}}^{2}\right)\right)}=\frac{1-2\theta }{2\theta \left(1-\theta \right)\log\left(\left(1-\theta \right)/\theta \right)}(1+o(1)). \end{array}$$

Define the function

$$\begin{array}{c} \tau (x)=\frac{1-2x}{2x(1-x)\log\left(\left(1-x\right)/x\right)},\;g(x)=\left(-2x^{2}+2x-1\right)\log\left(\frac{1}{x}-1\right)-2x+1 \end{array}$$

for 0<x<1/2 . Some algebra gives that

$$\begin{array}{c} \tau^{\prime }\left(x\right)=\frac{g(x)}{2x^{2}{\left(1-x\right)}^{2}\log^{2}\left(\frac{1}{x}-1\right)}. \end{array}$$

Note that

$$\begin{array}{c} g^{\prime }(x)=\frac{{\left(1-2x\right)}^{2}}{x(1-x)}+2\left(1-2x\right)\log\left(\frac{1}{x}-1\right)>0, \end{array}$$

for 0<x<1/2 , which implies that $g(x)<\lim_{x\to \frac{1}{2}}g(x)=0$ . Hence, we have $\tau^{\prime }(x)<0$ and $\tau (x)>\lim_{x\to \frac{1}{2}}\tau (x)=1$ . Thus, we have

$$\begin{array}{c} \frac{-\log\left(\sqrt{\theta_{j,k_{1}}\theta_{j,k_{2}}}+\sqrt{\left(1-\theta_{j,k_{1}}\right)\left(1-\theta_{j,k_{2}}\right)}\right)}{{\left(\theta_{j,k_{1}}-\theta_{j,k_{2}}\right)}^{2}/\left(8\left(\sigma_{\theta_{j,k_{1}}}^{2}\vee \sigma_{\theta_{j,k_{1}}}^{2}\right)\right)}=\tau (\theta )(1+o(1))>1, \end{array}$$

where τ(θ) is monotone decreasing as θ increases to 1/2 and τ(θ)→∞ as θ→0 .

The analysis for case (ii) is essentially the same and hence omitted. Combining (i) and (ii), we can arrive at

$$\begin{array}{c} I_{k_{1},k_{2}}=\sum_{j\in \Omega_{0}\left(k_{1},k_{2}\right)}I\left(\theta_{j,k_{1}},\theta_{j,k_{2}}\right)+\sum_{j\in \Omega_{0}^{c}\left(k_{1},k_{2}\right)}I\left(\theta_{j,k_{1}},\theta_{j,k_{2}}\right) \end{array}$$

and

\exp−Ik1,k22=\exp−12∑j∈Ω0k1,k2Iθj,k1,θj,k2\exp−12∑j∈Ω0ck1,k2Iθj,k1,θj,k2≳\exp−∑j∈Ω0k1,k2Δj,k1,k22⋅Iθj,k1,θj,k2/2Δj,k1,k22≳\exp−∑j∈Jθj,k1−θj,k228^¯σ21+τk1,k2,

where the inequality holds due to the assumption $\left|\Omega_{0}^{c}\right|=O(1)$ , and

$$\begin{array}{c} \tau_{k_{1},k_{2}}:=\frac{\sum_{j\in \Omega_{0}\left(k_{1},k_{2}\right)}\Delta_{j,k_{1},k_{2}}^{2}\cdot \tau \left(\theta_{j,k_{1}}\right)}{\sum_{j\in \Omega_{0}\left(k_{1},k_{2}\right)}\Delta_{j,k_{1},k_{2}}^{2}}\left(1+o(1)\right). \end{array}$$

### B.6 Proof of Lemma 1

In light of Corollary 3.12, Remark 3.13 in Bandeira and Van Handel (2016), and Theorem 6.10 in Boucheron et al. (2013), for t≥0, we have

$$\begin{array}{c} P\left(\left\|E\right\|\geq \frac{(1+\varepsilon )\sqrt{2}}{2}\left(\sqrt{N}+\sqrt{J}\right)+C_{\varepsilon }\sqrt{\log\left(N\wedge J\right)}+t\right)\leq (N\wedge J)e^{-t^{2}/2}. \end{array}$$

By choosing $t=2\sqrt{\log(N\wedge J)}$ and $\varepsilon =\sqrt{2}-1$ , for N,J large enough, we obtain that

$$\begin{array}{c} P\left(\left\|E\right\|\geq 2\left(\sqrt{N}+\sqrt{J}\right)\right)\leq {\left(N\wedge J\right)}^{-1}. \end{array}$$

This implies that $\lambda_{K+1}\left(R\right)\leq \left\|E\right\|\leq 2\left(\sqrt{J}+\sqrt{N}\right)$ with probability 1−o(1) . On the other hand, by assumption in Proposition 1 on $\sigma_{K}(ER)$ , we obtain that

$$\begin{array}{c} \lambda_{K}\left(R\right)\geq \lambda_{K}\left(ER\right)-\left\|E\right\|=\omega \left(\sqrt{N}+\sqrt{J}\right) \end{array}$$

with probability 1−o(1) . This completes the proof of Lemma 1.

### B.7 Special case when there exists $\theta_{j,k}$ close to 1/2

For θ<1/2 , we have

$$\begin{array}{c} \sigma_{\theta }^{2}=\frac{1-2\theta }{2\log\left(\left(1-\theta \right)/\theta \right)}=\left(\frac{1}{2}-\theta \right)\frac{1}{\log\left(1+\frac{1}{\theta }-2\right)}\geq \frac{\theta }{2}. \end{array}$$

For θ>1/2 , we have

$$\begin{array}{c} \sigma_{\theta }^{2}=\frac{2\theta -1}{2\log\left(\theta /\left(1-\theta \right)\right)}=\left(\theta -\frac{1}{2}\right)\frac{1}{\log\left(1+\frac{2\theta -1}{1-\theta }\right)}\geq \frac{1-\theta }{2}. \end{array}$$

Denote $\bar{\theta }:=\backslash operatorname*{\text{argmin}}_{\theta \in \left.l,b,r,a,ce\theta_{j,k},j\in J,k\in K,r,b,r,a\right.ce}\left|\frac{1}{2}-\theta \right|$ and $\bar{\delta }:=\left|\frac{1}{2}-\bar{\theta }\right|$ , then we simply have

^¯σ2=σθ¯2≥θ¯∧1−θ¯2=141−2δ¯.

We thereby have

18^¯σ2θj,k1−θj,k22≤121−2δ¯θj,k1−θj,k22.

We have $\bar{\delta }=o(1)$ by assumption, and without loss of generality assume $\frac{1}{2}>\theta_{j,k_{1}}>\theta_{j,k_{2}}$ and denote $\theta :=\theta_{2}$ , $\delta :=\theta_{1}-\theta_{2}=o(1)$ . Then we have

θj,k1−θj,k228^¯σ2=δ221+o(1).

On the other hand, we have

$$\begin{array}{cccc} -\log\left(\sqrt{\theta_{j,k_{1}}\theta_{j,k_{2}}}+\sqrt{\left(1-\theta_{j,k_{1}}\right)\left(1-\theta_{j,k_{2}}\right)}\right) & =\frac{\left(1+\bar{\_}C\theta +\delta ,\theta \right)}{2}{\left(\sqrt{\theta +\delta }-\sqrt{\theta }\right)}^{2}\left(1+o(1)\right) & =\delta^{2}\frac{\left(1+\bar{\_}C\theta +\delta ,\theta \right)}{8\theta }\left(1+o(1)\right) & =\frac{\delta^{2}}{8\theta \left(1-\theta \right)}\left(1+o(1)\right), \end{array}$$

where we’ve used the fact that $\bar{\_}C\theta +\delta ,\theta =\frac{\theta }{1-\theta }(1+o(1)).$ This implies that

−logθj,k1θj,k2+1−θj,k11−θj,k2θj,k1−θj,k22/8^¯σ2=14θ1−θ(1+o(1))>1,

where the last inequality holds due to θ<1/2 .

## C Proofs of lemmas

### C.1 Proof of Lemma B.1

To keep notation simple in the analysis, for any $s\in K^{N}$ , we define

$$\begin{array}{c} \theta_{j,k}\left(s\right):=\frac{\sum_{i\in N}R_{i,j}I\left(s_{i}=k\right)}{\sum_{i\in N}I\left(s_{i}=k\right)},\;j\in J,k\in K. \end{array}$$

The following result is needed, whose proof is deferred to Section C.2. Lemma C.1. Suppose Assumptions 1 and 2 hold. For any $\gamma_{0}>0$ , there exist some absolute constants $C_{1},C_{2}>0$ depending only on $\gamma_{0}$ such that with probability at least $1-2JKN^{-\left(2\gamma_{0}+1\right)}$ ,

(C.1)
$$\begin{array}{c} \max_{s:\ell_{0}\left(s,s^{⋆}\right)\leq \alpha /\left(2K\right)}\max_{j\in J,k\in K}\frac{\left|\theta_{j,k}\left(s\right)-\theta_{j,k}\left(s^{⋆}\right)\right|}{\ell_{0}\left(s,s^{⋆}\right)}\leq \frac{C_{1}K}{\alpha }\left(1+\bar{\sigma }\sqrt{\frac{K\log N}{\alpha N}}\right), \end{array}$$

and

(C.2)
$$\begin{array}{c} \max_{j\in J,k\in K}\left|\theta_{j,k}\left(s^{⋆}\right)-\theta_{j,k}\right|>C_{2}\bar{\sigma }\sqrt{\frac{K\log N}{\alpha N}}, \end{array}$$

where $\ell_{0}\left(s,s^{\prime }\right):=\frac{1}{N}\sum_{i\in N}I\left(s_{i}\neq s_{i}^{\prime }\right)$ . As a result, for any (possibly random) $s\in K^{N}$ such that $\ell_{0}(s,s^{⋆})\leq \alpha /(2K)$ , we have

$$\begin{array}{c} \max_{j\in J,k\in K}\left|\theta_{j,k}\left(s\right)-\theta_{j,k}\right|\leq \ell_{0}\left(s,s^{⋆}\right)\cdot \frac{C_{1}K}{\alpha }\left(1+\bar{\sigma }\sqrt{\frac{K\log N}{\alpha N}}\right)+C_{2}\bar{\sigma }\sqrt{\frac{K\log N}{\alpha N}} \end{array}$$

with probability at least $1-2JKN^{-(2\gamma_{0}+1)}$ .

By Proposition 1 and Markov’s inequality, we obtain that if

$$\begin{array}{c} \min_{\backslash lbra}ce\frac{\Delta /\bar{\sigma }}{K\left(1+\sqrt{\frac{J}{N}}\right)},\frac{\lambda_{K}/\bar{\sigma }}{\left(\sqrt{N}+\sqrt{J}\right)}\backslash rbrace\to \infty , \end{array}$$

then for m∈2 , with probability exceeding at least 1−\exp(−(1−c0)Δ2/(8^¯σ)) , we have

ℓs(m)~,sSm⋆≤\exp−1−c0Δ28^¯σ2,

where $s_{S_{m}}^{⋆}$ is sub-vector of $s^{⋆}$ with the index set $S_{m}$ , and we’ve used that \exp(−N/2)<\exp(−(1−c0)Δ2/(8^¯σ2)) . Without loss of generality, we assume $\ell \left(\tilde{s^{\left(m\right)}},s_{S_{m}}^{⋆}\right)=\ell_{0}\left(\tilde{s^{\left(m\right)}},s_{S_{m}}^{⋆}\right)$ . Combined with Lemma C.1, we then have

maxj∈J,k∈Kθj,k(m)^−θj,k≤C1\exp−1−c0Δ28^¯σ2+C2σ¯KlogNN

with probability at least 1−\exp−1−c0Δ2/8^¯σ2−\exp−CN/K−N−1+γ0 , provided that there exists some universal constant $\gamma_{0}>0$ such that

$$\begin{array}{c} JK≲N^{\gamma_{0}},\;N≳K\log N. \end{array}$$

The proof is completed by noticing that I⋆≍Δ2/^¯σ2 , JK≤\exp1−c1Δ2/8^¯σ2 for some $c_{1}\in (c_{0},1)$ , $\bar{\sigma }≍1,$ and

$$\begin{array}{c} \frac{\Delta }{J^{1/2}K^{3/4}{\left(\log N\right)}^{1/4}/N^{1/4}}\to \infty . \end{array}$$

### C.2 Proof of Lemma C.1

We introduce the following notations:

$$\begin{array}{c} \bar{\_}\theta j,k\left(s\right):=\frac{\sum_{i\in N}\theta_{i,s_{i}^{⋆}}I\left(s_{i}=k\right)}{\sum_{i\in N}I\left(s_{i}=k\right)},\;E_{j,k}(s):=\frac{\sum_{i\in N}E_{i,j}I\left(s_{i}=k\right)}{\sum_{i\in N}I\left(s_{i}=k\right)}. \end{array}$$

By definition, we have $\bar{\_}\theta j,k\left(s^{⋆}\right)=\theta_{j,k}$ and $E_{j,k}(s)=\theta_{j,k}\left(s\right)-\bar{\_}\theta j,k\left(s\right)$ , and hence

(C.3)
$$\begin{array}{cccc} & \left|\theta_{j,k}\left(s\right)-\theta_{j,k}\right| & =\left|\theta_{j,k}\left(s\right)-\bar{\_}\theta j,k\left(s\right)+\bar{\_}\theta j,k\left(s\right)-\bar{\_}\theta j,k\left(s^{⋆}\right)+\bar{\_}\theta j,k\left(s^{⋆}\right)-\theta_{j,k}\left(s^{⋆}\right)+\theta_{j,k}\left(s^{⋆}\right)-\theta_{j,k}\right| & \leq \left|E_{j,k}(s)-E_{j,k}\left(s^{⋆}\right)\right|+\left|\bar{\_}\theta j,k\left(s\right)-\bar{\_}\theta j,k\left(s^{⋆}\right)\right|+\left|\theta_{j,k}\left(s^{⋆}\right)-\theta_{j,k}\right|. \end{array}$$

We first bound the term $\left|E_{j,k}(s)-E_{j,k}\left(s^{⋆}\right)\right|$ . Notice that

(C.4)
$$\begin{array}{ccccc} & \left|E_{j,k}(s)-E_{j,k}\left(s^{⋆}\right)\right| & \leq \left|\frac{\sum_{i\in N}E_{i,j}I\left(s_{i}=k\right)}{\sum_{i\in N}I\left(s_{i}=k\right)}-\frac{\sum_{i\in N}E_{i,j}I\left(s_{i}^{⋆}=k\right)}{\sum_{i\in N}I\left(s_{i}=k\right)}\right|+\left|\frac{\sum_{i\in N}E_{i,j}I\left(s_{i}^{⋆}=k\right)}{\sum_{i\in N}I\left(s_{i}=k\right)}-\frac{\sum_{i\in N}E_{i,j}I\left(s_{i}^{⋆}=k\right)}{\sum_{i\in N}I\left(s_{i}^{⋆}=k\right)}\right| & \leq \frac{1}{\sum_{i\in N}I\left(s_{i}=k\right)}\left(\left|\sum_{i\in N}E_{i,j}I\left(s_{i}=k,s_{i}^{⋆}\neq k\right)\right|+\left|\sum_{i\in N}E_{i,j}I\left(s_{i}\neq k,s_{i}^{⋆}=k\right)\right|\right) & +\left|\frac{\sum_{i\in N}\left[I\left(s_{i}^{⋆}=k\right)-I\left(s_{i}=k\right)\right]}{\sum_{i\in N}I\left(s_{i}=k\right)}\right|\left|\frac{\sum_{i\in N}E_{i,j}I\left(s_{i}^{⋆}=k\right)}{\sum_{i\in N}I\left(s_{i}^{⋆}=k\right)}\right|. \end{array}$$

Note that the following fact holds for any $s\in K^{N}$ such that $\ell \left(s,s^{⋆}\right)\leq \alpha /\left(2K\right)$ :

$$\begin{array}{c} \sum_{i\in N}I\left(s_{i}=k\right)\geq \sum_{i\in N}I\left(s_{i}^{⋆}=k\right)-\sum_{i\in N}I\left(s_{i}^{⋆}\neq s_{i}\right)\geq \frac{\alpha N}{K}-\frac{\alpha N}{2K}\geq \frac{\alpha N}{2K}. \end{array}$$

Combined with the fact that $\left|E_{i,j}\right|\leq 1$ , we thus obtain the following bound for the first term in (C.4):

$$\begin{array}{c} \frac{1}{\sum_{i\in N}I\left(s_{i}=k\right)}\left(\left|\sum_{i\in N}E_{i,j}I\left(s_{i}=k,s_{i}^{⋆}\neq k\right)\right|+\left|\sum_{i\in N}E_{i,j}I\left(s_{i}\neq k,s_{i}^{⋆}=k\right)\right|\right)\leq \frac{4K}{\alpha }\ell \left(s,s^{⋆}\right). \end{array}$$

Next, we bound the second term in (C.4). By General Hoeffding’s inequality, we obtain that

P∑i∈NEi,jIsi⋆=k∑i∈NIsi⋆=k≥t≤2\exp−ct2^¯σ2Nk.

By choosing $t=\bar{\sigma }\sqrt{CN_{k}\log N}$ for some sufficiently large C>0 , we have that

$$\begin{array}{c} \left|\frac{\sum_{i\in N}E_{i,j}I\left(s_{i}^{⋆}=k\right)}{\sum_{i\in N}I\left(s_{i}^{⋆}=k\right)}\right|\leq C\bar{\sigma }\sqrt{\frac{K\log N}{\alpha N}} \end{array}$$

with probability exceeding $1-N^{-2\gamma_{0}-1}$ for some large constant $\gamma_{0}>0$ . In addition, we have

$$\begin{array}{ccc} \left|\frac{\sum_{i\in N}\left[I\left(s_{i}^{⋆}=k\right)-I\left(s_{i}=k\right)\right]}{\sum_{i\in N}I\left(s_{i}=k\right)}\right| & \leq \frac{2K}{\alpha N}\left(\left|\sum_{i\in N}I\left(s_{i}^{⋆}=k,s_{i}\neq k\right)\right|+\left|\sum_{i\in N}I\left(s_{i}^{⋆}\neq k,s_{i}=k\right)\right|\right) & ≲\frac{K}{\alpha }\ell \left(s,s^{⋆}\right). \end{array}$$

From (C.4) and a union bound over j∈J and k∈K , we arrive at

$$\begin{array}{c} \max_{j\in J,k\in K}\left|E_{j,k}(s)-E_{j,k}\left(s^{⋆}\right)\right|≲\alpha^{-1}K\ell \left(s,s^{⋆}\right)\left(1+\bar{\sigma }\sqrt{\frac{K\log N}{\alpha N}}\right) \end{array}$$

with probability at least $1-JKN^{-2\gamma_{0}-1}$ .

Next, we bound the term $\left|\bar{\_}\theta j,k\left(s\right)-\bar{\_}\theta j,k\left(s^{⋆}\right)\right|$ . Notice that by definition $\bar{\_}\theta j,k\left(s^{⋆}\right)=\theta_{j,k}$ , hence

$$\begin{array}{c} \left|\bar{\_}\theta j,k\left(s\right)-\bar{\_}\theta j,k\left(s^{⋆}\right)\right|=\left|\frac{\sum_{i\in N}\sum_{k^{\prime }\in K}\left(\theta_{i,k}-\theta_{i,k^{\prime }}\right)I\left(s_{i}=k,s_{i}^{⋆}=k^{\prime }\right)}{\sum_{i\in N}I\left(s_{i}=k\right)}\right|\leq \frac{2K}{\alpha }\ell \left(s,s^{⋆}\right). \end{array}$$

It suffices to bound $\left|\theta_{j,k}\left(s^{⋆}\right)-\theta_{j,k}\right|$ by

$$\begin{array}{c} \max_{j\in J,k\in K}\left|\theta_{j,k}\left(s^{⋆}\right)-\theta_{j,k}\right|=\max_{j\in J,k\in K}\left|\frac{\sum_{i\in N}E_{i,j}I\left(s_{i}^{⋆}=k\right)}{\sum_{i\in N}I\left(s_{i}^{⋆}=k\right)}\right|\leq C\bar{\sigma }\sqrt{\frac{K\log N}{\alpha N}} \end{array}$$

with probability exceeding $1-JKN^{-2\gamma_{0}-1}$ .

Collecting (C.3) and the bounds for $\left|E_{j,k}(s)-E_{j,k}\left(s^{⋆}\right)\right|$ , $\left|\bar{\_}\theta j,k\left(s\right)-\bar{\_}\theta j,k\left(s^{⋆}\right)\right|$ , and $\left|\theta_{j,k}\left(s^{⋆}\right)-\theta_{j,k}\right|$ , we can complete the proof.

## Figure 1 Long description

The left panel displays three individual points spread across the plot, each at distinct positions along Dimension 1 and Dimension 2. The right panel shows three compact horizontal clusters of points, each cluster aligned at a different value along Dimension 2 but sharing similar values along Dimension 1. Both panels have axes labeled Dimension 1 on the x-axis and Dimension 2 on the y-axis, with ranges from negative twelve to negative six for Dimension 1 and negative six to six for Dimension 2.

Navigate back to Figure 1.

## Algorithm 1: Long description

The heading states Algorithm 1: Spectral clustering (Spec). The data matrix R is in R to the N times J, with K latent classes. The result is estimated latent class labels s tilde in open bracket K close bracket to the N. Step 1: Perform rank-K S V D on R to obtain the summation from i equals 1 to K of lambda i times u hat i times v hat i transpose, where lambda 1 is greater than or equal to lambda 2 and so on, greater than or equal to lambda K, greater than or equal to zero. U hat equals open parenthesis u 1, dot dot dot, u K close parenthesis in R to the N times K, and V hat equals open parenthesis v 1, dot dot dot, v K close parenthesis in R to the J times K. Step 2: Perform K-means on the rows of U hat Sigma, or equivalently R V hat, i.e., open parenthesis s tilde, c tilde k for k in open bracket K close bracket close parenthesis equals arg min over s in open bracket K close bracket to the N, c tilde k for k in open bracket K close bracket in R to the K, of the summation over i in open bracket N close bracket of the norm squared of U hat i, Sigma minus c s i.

Navigate back to Algorithm 1:.

## Algorithm 2: Long description

The algorithm is titled ‘Algorithm 2: SOLA.’ The data input is a matrix R in the set of real numbers to the N by J, with rank K. The result is estimated labels s-bar in the set of K to the N. Step 1 initializes label estimates as s-tilde equals Spec of R. Step 2, labeled Maximization, computes initial center estimates: theta-bar sub j,k equals the summation over i in N of R sub i,j times the indicator function that s-tilde sub i equals k, all over the summation over i in N of the indicator function that s-tilde sub i equals k, for j in J and k in K. This is equation (7). Step 3, labeled Assignment, performs one-step likelihood-based refinement: s-bar sub i equals the argument maximum over k in K of the sum over j in J of R sub i,j times the logarithm of theta-bar sub j,k plus the quantity one minus R sub i,j times the logarithm of one minus theta-bar sub j,k, for i in N. This is equation (8).

Navigate back to Algorithm 2:.

## Algorithm 3: Long description

The flowchart begins with the title Algorithm 3 SOLA with sample splitting. Step 1 defines the data matrix R as a binary matrix of size N by J, index sets S sub 1 and S sub 2, and number of clusters K. The result is the estimated labels s hat superscript E, a vector of length N with entries in K. Step 1 instructs to construct sub-matrices R sub S sub 1 and R sub S sub 2 from R using the index sets. Step 2 initializes label estimates as s hat superscript m equals Spec of R sub S sub m, for m equals 1 and 2. Step 3 defines initial center estimates as theta hat sub j k superscript m equals the sum over i in S sub m of R sub i j times the indicator that s hat sub i superscript m equals k, all over the sum over i in S sub m of the indicator that s hat sub i superscript m equals k, for all j in J, k in K, m equals 1, 2. Step 4 performs one-step likelihood-based refinement, updating s tilde sub i superscript l as the argument over k in K that maximizes the sum over j in J of R sub i j times log of theta hat sub j k superscript m plus one minus R sub i j times log of one minus theta hat sub j k superscript m, for i in S sub l, l not equal to m in 1, 2. Equation 16 is referenced here. Step 5 aligns labels by setting s tilde sub i equals s tilde sub i superscript 1 for i in S sub 1. For S sub 2, pi hat is defined as the permutation in S sub K that minimizes the Frobenius norm of theta hat superscript 1 minus theta hat superscript 2 times G sub pi, where S sub K is the set of all permutations of K and G sub pi is the column permutation matrix for pi. The final labels for S sub 2 are set as pi hat of s tilde sub i superscript 2.

Navigate back to Algorithm 3:.

## Table 1 Long description

The table has four columns for methods: S O L A, S O L A plus, E M by polca, and E M by LatentGold. Three rows represent different settings. First row, N equals 190, J equals 95, labeled N equals 2 J: S O L A 0.0815, S O L A plus 0.0772, E M by polca 0.3022, E M by LatentGold 0.0645. Second row, N equals 95, J equals 95, labeled N equals J: S O L A 0.1760, S O L A plus 0.1745, E M by polca 0.4642, E M by LatentGold 0.2609. Third row, N equals 75, J equals 150, labeled N equals J all over 2: S O L A 0.0411, S O L A plus 0.0411, E M by polca 0.5002, E M by LatentGold 0.2795. Clustering error is lowest for S O L A and S O L A plus in all settings, highest for E M by polca.

Navigate back to Table 1.

## Figure 2 Long description

The x-axis is labeled Number of items J (sample size N equals 2 J), ranging from 20 to 100. The y-axis is labeled Misclustering Proportion, ranging from 0.1 to 0.5. Five methods are plotted: E M (red circles, solid line), S O L A (olive triangles, dashed line), S O L A plus (green squares, dashed line), S V D (blue plus signs, dash-dot line), and Tensor dash C E M (pink squares, dotted line). All methods start near 0.5 misclustering proportion at low J values. As J increases, all lines show a downward trend. E M and Tensor dash C E M decrease more gradually, while S O L A, S O L A plus, and S V D drop sharply and converge near 0.1 at high J values. The legend is positioned above the plot, with each method’s symbol and color clearly indicated.

Navigate back to Figure 2.

## Figure 3 Long description

The x-axis is labeled Number of items J with values from 30 to 90. The y-axis is labeled Misclustering Proportion, ranging from 0.1 to 0.5. Five methods are compared: E M (red circles, solid line), S O L A (olive triangles, dashed line), S O L A plus (green squares, dashed line), S V D (blue pluses, dashed line), and Tensor-C E M (magenta hollow squares, dotted line). All methods show a general decrease in misclustering proportion as J increases. E M starts near 0.5 and decreases to about 0.3, remaining consistently higher than the other methods. S O L A, S O L A plus, and S V D overlap closely, starting near 0.43 and dropping below 0.15 as J increases. Tensor-C E M starts at 0.5, decreases more sharply, and converges with the other non-E M methods for higher J. The legend is at the top, with method names and symbols.

Navigate back to Figure 3.

## Figure 4 Long description

The x-axis is labeled Number of items J (sample size N equals 2 J), ranging from 20 to 100. The y-axis is labeled Failure Rate, spanning 0.00 to 0.18. Five methods are plotted: E M (red circles), S O L A (green triangles), S O L A plus (green squares), S V D (blue plus signs), and Tensor-C E M (pink squares with dotted line). Tensor-C E M starts at the highest failure rate near 0.18 at J equals 20 and decreases sharply to below 0.02 by J equals 100. The other methods remain close to zero throughout, with minor fluctuations. The legend is positioned at the top, showing all method symbols and colors.

Navigate back to Figure 4.

## Table 2 Long description

The table has two rows for J values 25 and 95, and five columns for methods S V D, S O L A, S O L A plus, E M, and Tensor dash C E M. For J equals 25, running times are: S V D 4.15 times 10 to the minus 4, S O L A 8.62 times 10 to the minus 4, S O L A plus 4.19 times 10 to the minus 3, E M 1.42 times 10 to the minus 2, Tensor dash C E M 5.12 times 10 to the minus 1. For J equals 95, running times are: S V D 2.33 times 10 to the minus 3, S O L A 4.66 times 10 to the minus 3, S O L A plus 2.51 times 10 to the minus 2, E M 1.80 times 10 to the minus 1, Tensor dash C E M 6.95 times 10 to the minus 1. Running times increase for all methods as J increases, with Tensor dash C E M consistently the slowest and S V D the fastest.

Navigate back to Table 2.

## Figure 5 Long description

The box plot displays running time in seconds on the y-axis and five methods on the x-axis, ordered left to right as E M, S O L A, S O L A plus, S V D, and Tensor dash C E M. E M shows a median near 0.2 with outliers up to 0.4. S O L A, S O L A plus, and S V D have medians at zero with minimal spread and a few small outliers. Tensor dash C E M has the highest median near 0.7, with a tight interquartile range and outliers above 0.75. Red dots indicate outliers for each method.

Navigate back to Figure 5.

## Figure 6 Long description

The x axis shows Number of items J, ranging from 30 to 90, with sample size N equal to J. The y axis shows Misclustering Proportion from 0.2 to 0.5. Six methods are plotted: E M (red circles), S O L A (green squares), S O L A plus (brown triangles), S V D (blue squares), Spec dash E M (cyan plus signs), and Tensor dash C E M (magenta asterisks). E M starts near 0.45 and remains above 0.4 across all J. S O L A and S O L A plus drop sharply below 0.3 as J increases, reaching the lowest values near 0.2 at J equals 90. S V D, Spec dash E M, and Tensor dash C E M show intermediate performance, with Tensor dash C E M and S V D converging near 0.2 at high J. All methods except E M show decreasing misclustering proportion as J increases.

Navigate back to Figure 6.

## Figure 7 Long description

The x axis shows Number of items J, ranging from 80 to 140, with sample size N equals 0.5 J. The y axis shows Misclustering Proportion from 0.1 to 0.5. Six methods are plotted: E M (red circles), S O L A (green squares), S V D (blue crosses), S O L A plus (brown triangles), Spec-E M (cyan plus signs), and Tensor-C E M (pink stars). E M and Spec-E M maintain high misclustering proportions near 0.5 across all J values. S O L A, S V D, S O L A plus, and Tensor-C E M start at about 0.3 and decrease steadily to below 0.1 as J increases. All methods are labeled in the legend at the top left.

Navigate back to Figure 7.

## Figure 8 Long description

The x axis is labeled Number of items J, with sample size N equals 100, ranging from 100 to 250. The y axis is labeled Misclustering Proportion, ranging from 0.0 to 0.5. Six methods are plotted: E M (red circles), S O L A (green squares), S V D (blue crosses), S O L A plus (brown triangles), Spec-E M (cyan dashed line with plus signs), and Tensor-C E M (pink stars). E M maintains the highest misclustering proportion, around 0.45, across all J values. Spec-E M shows a moderate error rate, fluctuating near 0.33. S O L A, S V D, S O L A plus, and Tensor-C E M all start near 0.2 at J equals 100 and decrease steadily, converging below 0.05 by J equals 250. All methods except E M and Spec-E M show improved clustering accuracy as J increases.

Navigate back to Figure 8.

## Figure 9 Long description

The x axis displays sample size N ranging from 100 to 250, with J fixed at 100. The y axis shows misclustering proportion from 0 to 0.5. Six methods are plotted: E M (red circles) starts at 0.45 and decreases steadily to 0.25 as N increases. Spec-E M (cyan plus) begins near 0.4 and drops sharply to below 0.1. Tensor-C E M (magenta asterisk) starts at 0.2 and decreases to about 0.08. S O L A (green square), S O L A plus (brown triangle), and S V D (blue crosshatch) all begin near 0.1 and decrease slightly, converging below 0.08. All methods show decreasing misclustering with increasing sample size, with E M consistently highest and S O L A, S O L A plus, S V D lowest.

Navigate back to Figure 9.

## Figure 10 Long description

The x-axis is labeled Sample size N number of items J equals 30, ranging from 75 to 250 in increments of 25. The y-axis is labeled Misclustering Proportion, ranging from 0.35 to 0.50. Six methods are shown: E M red circles, S O L A green squares, S V D blue open squares, S O L A plus brown triangles, Spec-E M cyan plus signs, and Tensor-C E M magenta asterisks. All methods show a general decrease in misclustering proportion as N increases. Spec-E M consistently achieves the lowest misclustering proportions, dropping from about 0.42 at N equals 75 to below 0.35 at N equals 250. Tensor-C E M remains the highest, fluctuating around 0.48 to 0.50. E M starts near 0.47 and decreases to about 0.39. S O L A, S V D, and S O L A plus cluster between 0.40 and 0.37 at higher N. The legend appears at the top left, with method names and corresponding symbols and colors.

Navigate back to Figure 10.

## Figure 11 Long description

The x-axis shows Number of items J ranging from 30 to 90, with sample size N equal to J. The y-axis shows Misclustering Proportion from 0.2 to 0.5. Six methods are compared: E M (red circles), S O L A (green squares), S V D (blue crosses), S O L A plus (brown triangles), Spec-E M (cyan dashed line with pluses), and Tensor-C E M (magenta stars and dashed line). All methods start near 0.45 misclustering at J equals 30. As J increases, Tensor-C E M and Spec-E M show the steepest decline, reaching about 0.2 at J equals 90. E M remains highest, ending near 0.4. S O L A, S V D, and S O L A plus cluster together, declining to about 0.3 at J equals 90. The legend is at the top left.

Navigate back to Figure 11.

## Figure 12 Long description

The x-axis is labeled Number of items J with sample size N equals J, ranging from 20 to 100. The y-axis is labeled Misclustering Proportion, ranging from 0 to 0.35. Six methods are shown: E M (red circles, solid line), S O L A (green squares, solid line), S V D (blue open squares, solid line), S O L A plus (brown triangles, solid line), Spec-E M (cyan plus, solid line), and Tensor-C E M (magenta stars, dashed line). E M starts at the highest misclustering proportion near 0.33 at J equals 20 and decreases steadily to about 0.07 at J equals 100, always above the other methods. The other five methods cluster closely together, starting near 0.23 at J equals 20 and decreasing rapidly to below 0.05 by J equals 100. Tensor-C E M starts slightly higher than the other non-E M methods but converges with them as J increases. All methods show a decreasing trend in misclustering proportion as J increases.

Navigate back to Figure 12.

## Table 3 Long description

The table has four rows for item parameter mechanisms and six columns for J values: one thousand, one thousand two hundred, one thousand four hundred, one thousand six hundred, one thousand eight hundred, two thousand. The first row, Fixed design of item parameters, shows one hundred percent for all J values. The second row, theta sub j k i dot i dot d dot sim Beta one one, also shows one hundred percent for all J values. The third row, theta sub j k i dot i dot d dot sim Beta one four, shows zero percent for one thousand and one thousand two hundred, then one hundred percent for one thousand four hundred and higher. The fourth row, theta sub j k i dot i dot d dot sim Beta one five, shows zero percent for one thousand through one thousand six hundred, seventy percent for one thousand eight hundred, and one hundred percent for two thousand. The table demonstrates that the proportion of correct K selection increases with J for Beta one four and Beta one five, but remains perfect for Fixed and Beta one one designs.

Navigate back to Table 3.

## Table 4 Long description

The table has five columns for methods S V D, S O L A, S O L A plus, E M, and Tensor-C E M. The first row under the header lists mis-clustering error values: S V D 0.0212, S O L A 0.0212, S O L A plus 0.0212, E M 0.117, Tensor-C E M 0.0212. The second row lists running times in seconds: S V D 0.00179, S O L A 0.0344, S O L A plus 0.0503, E M 3.125, Tensor-C E M 5.154. All methods except E M achieve the lowest error of 0.0212, while S V D is the fastest and Tensor-C E M is the slowest.

Navigate back to Table 4.
